# Meta‐Analysis: Chronic Gastrointestinal Symptoms and Comorbidities in Hypermobile Ehlers–Danlos Syndrome and Hypermobility Spectrum Disorders

**DOI:** 10.1111/apt.70856

**Published:** 2026-07-12

**Authors:** Dmitrii Kulin, Gerald Holtmann, Thomas Fairlie, Kyle Staller, Samuel Nurko, Laurie Keefer, Douglas A. Drossman, Michael P. Jones, Qasim Aziz, Ayesha Shah

**Affiliations:** ^1^ Faculty of Medicine The University of Queensland Brisbane Queensland Australia; ^2^ Translational Research Institute Brisbane Queensland Australia; ^3^ Department of Gastroenterology & Hepatology Princess Alexandra Hospital Brisbane Queensland Australia; ^4^ Centre for Neurointestinal Health, Division of Gastroenterology Massachusetts General Hospital Boston Massachusetts USA; ^5^ Harvard Medical School Boston Massachusetts USA; ^6^ Centre for Motility and Functional Gastrointestinal Disorders Boston Children's Hospital Boston Massachusetts USA; ^7^ Department of Pediatrics Harvard Medical School Boston Massachusetts USA; ^8^ Division of Gastroenterology Icahn School of Medicine at Mount Sinai New York City New York USA; ^9^ Division of Gastroenterology University of N.C. and Drossman Gastroenterology Chapel Hill North Carolina USA; ^10^ School of Psychological Sciences Macquarie University North Ryde Australia; ^11^ Wingate Institute of Neurogastroenterology, Queen Mary University of London London UK

**Keywords:** Ehlers–Danlos syndrome, gastrointestinal symptoms, hypermobility spectrum disorders, systematic review and meta‐analysis

## Abstract

**Background:**

Patients with Ehlers–Danlos syndrome (EDS)/hypermobility spectrum disorders (HSD) report higher rates of chronic gastrointestinal (GI) symptoms, disorders of gut–brain interaction (DGBI), and extraintestinal comorbidities.

**Aims:**

We conducted a systematic review and meta‐analysis to assess the prevalence of chronic GI symptoms and comorbid conditions in hEDS/HSD.

**Methods:**

Electronic databases were searched until December 2025 for studies reporting on chronic GI symptoms in hEDS/HSD patients. Pooled prevalence rates, odds ratios (ORs), and 95% confidence intervals (CIs) were calculated using the random effects model.

**Results:**

The final dataset of 19 studies included 17,455 hEDS/HSD patients and 1,677,465 controls. The odds of chronic GI symptoms were higher in patients with hEDS/HSD compared to controls (OR 4.29, 95% CI 3.1–6.0). 65.3% (95% CI 51.4–77.0) of hEDS/HSD patients reported at least one chronic GI symptom, with heartburn being the most common (34.7%, 95% CI 28.3–41.7). The prevalence of DGBI in hEDS/HSD patients was 44.2% (95% CI 23.9–66.6), with functional dysphagia the most common DGBI at 34.2% (95% CI 25.7–43.8). Gastroesophageal reflux disease was reported in 41.3% (95% CI 27.2–57.0). In hEDS/HSD patients, the most common extraintestinal comorbidity was chronic fatigue (49%, 95% CI 34.6–63.6), followed by migraine (38.2%, 95% CI 19.9–60.5), orthostatic intolerance (OI) (35.9%, 95% CI 26.6–46.4), fibromyalgia (27.9%, 95% CI 16.0–44.0) and postural orthostatic tachycardia syndrome (POTS) (21.9%, 95% CI 5.2–59.1).

**Conclusions:**

Overall, > 60% of hEDS/HSD patients report chronic GI symptoms. DGBI, co‐morbidities, including POTS, were highly prevalent in hEDS/HSD patients. However, the quality of the evidence is low due to significant clinical heterogeneity observed in the analyses. While the associations may suggest a causal relationship, the results should be interpreted with caution.

## Graphical abstract

This meta‐analysis reveals that a significant proportion of patients with hEDS/HSD experience gastrointestinal symptoms. The most prevalent symptoms include heartburn, abdominal pain, constipation, and nausea. There is a notable overlap of hEDS/HSD with chronic fatigue, DGBI, GERD, migraine, and orthostatic intolerance.

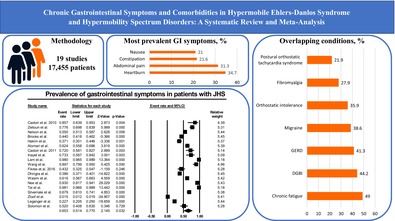

## 1 Introduction

The Ehlers–Danlos syndromes (EDS) is a heterogeneous group of 13 heritable non‐inflammatory connective tissue disorders characterized by joint hypermobility, skin hyperextensibility, and tissue fragility [1]. EDS is a multi‐system disorder and has been associated with several symptoms: neurological (e.g., headaches), cardiorespiratory (e.g., palpitations, chest pain, and dyspnoea), autonomic (e.g., syncope, postural instability, and thermoregulatory difficulties), urogynecological (e.g., prolapse, urinary incontinence, and dyspareunia), and gastroenterological (e.g., abdominal pain, postprandial fullness, constipation and diarrhoea) [2]. EDS is also associated with multiple co‐morbidities, including orthostatic intolerance (OI), organic gastrointestinal disorders and disorders of gut–brain interaction (DGBI), fibromyalgia, functional somatic syndromes [3, 4], psychiatric (e.g., mood disorders, anxiety, and sleep disturbances) and neurodevelopmental (Attention Deficit Hyperactivity Disorder [ADHD] and autism) disorders, and inflammatory and systemic manifestations related to mast cell activation [5].

Currently, a genetic defect can be identified in all EDS variants except hypermobile EDS (hEDS), which has no confirmatory laboratory test. Consequently, hEDS is the only EDS subtype with an unknown genetic basis and pathophysiology [1]. The diagnosis of hEDS is a clinical diagnosis—relying on physical examination, personal history, and the exclusion of other overlapping disorders [3]. In 2017, a revised nosology for hEDS was introduced. Patients who do not meet the updated criteria for EDS but exhibit symptoms of joint hypermobility are diagnosed with hypermobility spectrum disorder (HSD). HSD, previously known as joint hypermobility syndrome or EDS type III, is defined as musculoskeletal symptoms in a hypermobile individual in the absence of systemic rheumatologic disease, a population that was previously categorized under joint hypermobility [6]. These individuals often experience symptoms and co‐morbidities similar to those diagnosed with hEDS [1]. EDS is rare or ultra‐rare for several subtypes, but hEDS and HSD are much more common [7, 8]. It is estimated that the prevalence of HSD/hEDS is greater than 1:500 [8], and hEDS represents 80%–90% of the total burden of EDS [3]. Given the historical complexity of nomenclature and the predominance of hEDS in the EDS population, and to incorporate data that have used prior terminology, we shall further refer to this group as hEDS/HSD for the purposes of this meta‐analysis.

Several comorbidities are associated with hEDS/HSD. These include disorders of autonomic function, gastrointestinal function, neurodevelopment, mental health, and inflammation and systemic manifestations of mast cell activation [5]. Several studies have reported a high prevalence of chronic gastrointestinal symptoms consistent with DGBI in patients with hEDS/HSD [9, 10, 11]. A recent study [12] found that certain subgroups within hEDS/HSD have a high prevalence of functional foregut disorders and irritable bowel syndrome (IBS). These subgroups also face increased rates of psychological disorders, dysautonomia, and lower quality of life compared to those with hEDS/HSD alone. These are associations, and the pathological link between DGBI and hEDS/HSD remains to be established. In a large case–control study, 98% of subjects with hEDS/HSD fulfilled symptom‐based criteria for 1 or more Rome IV DGBI, compared with 47% of controls (*p* < 0.001) and had high healthcare utilization and considerable health impairment [9, 13].

Against this background, we performed a systematic review and meta‐analysis to assess the prevalence of chronic gastrointestinal symptoms in patients with hEDS/HSD compared to controls, as well as identifying the most common gastrointestinal symptom within this patient population. Our secondary objectives involved assessing the prevalence of (a) commonly occurring gastrointestinal comorbidities, including DGBI, in patients with hEDS/HSD, and (b) extraintestinal comorbidities in patients with hEDS/HSD.

## 2 Materials and Methods

This systematic review and meta‐analysis meet the preferred reporting items for systematic reviews and meta‐analysis statement requirements (PRISMA) [14, 15]. The protocol for this Systematic Review was prospectively registered with PROSPERO (registration number CRD420251269947).

### 2.1 Search Strategy

**FIGURE 1:**
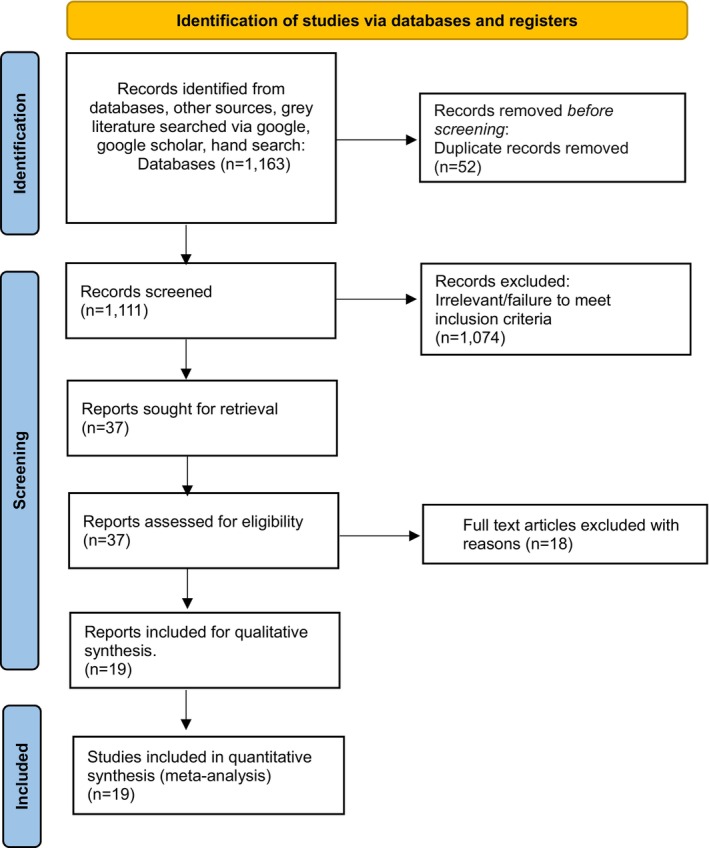
PRISMA flow diagram.

A comprehensive search of electronic databases, including MEDLINE (PubMed), EMBASE Cochrane, Web of Science and Scopus were conducted from 1 January 1952, until 1 December 2025 for all studies reporting on both adult and paediatric patients with HSD and gastrointestinal symptoms. The detailed literature search strategy is outlined in the PRISMA flow diagram (Figure 1) and was conducted with the assistance of a librarian. The search strategy for MEDLINE has been outlined in Table S1. The initial search was not limited to specific languages to capture all appropriate studies. Grey literature was searched with Google and Google Scholar, and the ‘Snowball’ method was also utilized to identify all relevant articles.

### 2.2 Selection of Studies

Two authors (D.K and T.F) independently conducted an initial screening of abstracts and titles. Abstracts were eliminated if they did not meet the inclusion criteria. Full texts of the remaining articles were retrieved and reviewed. The studies that were excluded are provided in Table S2.

Eligible studies included cross‐sectional studies reporting the prevalence of chronic GI symptoms in unselected subjects with hEDS/HSD, and case–control studies comparing chronic GI symptoms between unselected hEDS/HSD subjects and controls. We included only those studies where the diagnosis of hEDS/HSD was based on the clinical assessment or specific symptom‐based criteria, including Brighton criteria, Beighton score, and 2017 International criteria for hEDS/HSD. Studies not reporting on original data, those reporting on mixed populations of DGBI and subjects with organic gastrointestinal diseases, studies with no separate data on patients with hEDS/HSD or those not reporting on the prevalence of chronic symptoms in patients with HSD, as well as studies focusing on the prevalence of hEDS/HSD in patients with gastrointestinal conditions, were excluded. Conference abstracts [16] that provided sufficient data to meet the inclusion criteria were also included in the study in order to increase the comprehensiveness and precision of the analysis and minimize publication bias. Disagreements between reviewers were resolved by mutual consensus.

### 2.3 Data Extraction and Quality Assessment

Extracted data were entered into a Microsoft Excel spreadsheet (2024 Professional edition: Microsoft Corp, Redmond, Washington, USA). The following data were extracted: author, year of study, study design, country, mean age, gender, diagnostic criteria, inclusion/exclusion criteria of study, the number of patients with hEDS/HSD and chronic gastrointestinal symptoms, the number of patients with overlapping conditions, both gastrointestinal and extraintestinal.

The quality of the prevalence studies included was assessed using the Joanna Briggs Institute (JBI) critical appraisal tools for use in JBI systematic reviews for prevalence studies [17] (Table S4), and Newcastle‐Ottawa scale (NOS) for case–control studies [18] (Table S5). For the JBI critical appraisal tool, the risk of bias was ranked as high when the study reached up to 49% of ‘yes’ score, or had serious methodological flaws with decisive impact on validity, moderate when the study reached from 50% to 69% of ‘yes’ score with no such flaws, and low when the study reached over 70% of ‘yes’ score with no such flaws, thus combining the quantitative and qualitative approach to the risk of bias assessment. For NOS, the risk of bias was considered high when the study reached < 4 stars, moderate when the study reached 4–6 stars, and low when the study reached 7 or more stars.

### 2.4 Data Synthesis and Statistical Analysis

In an initial step, number of patients with hEDS/HSD with gastrointestinal symptoms were extracted, along with other items needed to calculate prevalence and to conduct subgroups analyses. It is worthy of note that eight studies included multiple EDS subtypes. As a second step, the pooled prevalence of chronic gastrointestinal symptoms in patients with hEDS/HSD was calculated. Separate analyses were performed to determine the pooled prevalence of individual gastrointestinal symptoms in patients with hEDS/HSD, other gastrointestinal and extraintestinal conditions in patients with hEDS/HSD, pooled prevalence of gastrointestinal symptoms in Ehlers–Danlos syndrome as the most frequently encountered hEDS/HSD within the included literature, and, finally, the odds ratio of the prevalence of DGBI in patients with hEDS/HSD compared to controls used in the studies with a control group. Analyses for individual symptoms (e.g., heartburn) and disorders presenting with those symptoms (e.g., functional heartburn) were carried out separately. Subgroup analyses comparing healthy controls versus patient controls and stratifying by geographic location were performed. Patient controls included other patients seen at the clinic with no history of a hEDS/HSD diagnosis.

Finally, we undertook a sensitivity analysis including only high‐quality studies (assessed utilizing the JBI critical appraisal tool), reporting the prevalence of gastrointestinal symptoms in patients with HSD, and a sensitivity analysis exploring the effects of outliers on the pooled prevalence. Analyses were carried out using the Comprehensive Meta‐Analysis (CMA) software (version 4) (Borenstein M., Hedges, L., Higgins, J., & Rothstein, H. Biostat, Englewood, NJ 2022). Logit transformation of proportions and the variance of the logit were used in the calculations to estimate pooled event rates and 95% confidence intervals (CIs) within groups and to compare event rates between groups. Pooled estimates of prevalence were estimated using the DerSimonian and Laird random effects model to account for systematic between‐study variability appropriately. For subgroup analyses with five or fewer studies we used the DerSimonian and Laird random effects analysis with the Knapp‐Hartung adjustment (Table S6); otherwise, no adjustment was employed in these models.

The proportion of total variability due to between‐study variation was evaluated using Cochrane's test and visual evaluation of forest plots and was quantified through the *I* ^2^ index. Further, we considered that either Cochrane *χ* ^2^ test *p* < 0.10 or *I* ^2^ > 50% indicated substantial heterogeneity, *I* ^2^ > 75% indicated high heterogeneity, and *I* ^2^ > 30% to *I* ^2^ < 60% indicated moderate heterogeneity. Heterogeneity was further explored via subgroup analysis. Assessment of publication bias in prevalence meta‐analyses remains challenging [19], as commonly used methods, including funnel plots and Egger's test, were developed for comparative effect measures. Therefore, standard approaches (Egger's test and visual inspection of funnel plots for evidence of asymmetry; standard error versus log[odds], sample size vs. log[odds]) were applied with caution to identify potential publication biases. Formal publication bias tests were only considered when at least 10 studies were available, due to the low reliability of these tests in analyses with fewer than 10 studies.

## 3 Results

### 3.1 Study Selection

The initial search revealed 1163 publications. Of these, 35 published articles appeared to be relevant to the study question and were retrieved for further evaluation. Out of those, 19 were eligible for inclusion in this systematic review and meta‐analysis, including one conference abstract (Figure 1 and Table S2). The characteristics of all the studies in the current meta‐analysis, including the methodology pertaining to diagnosis of hEDS/HSD and DGBI, patient characteristics, and geographic region are outlined in Table 1 and Table S3.

**TABLE 1:** Included study characteristics.

Study	Country (geographic region)	Study design	Control type	Total eligible participants, *n*	Total controls, *n*	hEDS/HSD patients with DGBI, *n*	hEDS/HSD patients with GI complaints, *n*	hEDS/HSD patients with OI, *n*
Castori et al. (2010) [20]	Italy (Europe)	Cross‐sectional	NA	21	NA	13	18	NA
Zeitoun et al. [21]	France (Europe)	Cross‐sectional	NA	134^a^	NA	64	104	NA
Nelson et al. [22]	USA (America)	Cross‐sectional	NA	687^a^	NA	189	378	251
Brooks et al. [23]	USA (America)	Case–control	Patients without EDS	2007^a^	4014	128	883	434
Hakim et al. [24]	UK (Europe)	Case–control	Healthy controls	170	50	NA	63	70
Alomari et al. [25]	USA (America)	Cross‐sectional	NA	218	NA	47	136	87
Castori et al. (2011) [26]	Italy (Europe)	Cross‐sectional	NA	50	NA	NA	36	39
Inayet et al. [27]	UK (Europe)	Case–control	Fracture clinic patients	45	45	27	33	NA
Lam et al. [9]	UK (Europe)	Case–control	Healthy controls	603	603	591	591	NA
Wang et al. [28]	USA (America)	Cross‐sectional	NA	68^a^	NA	6	61	6
Fikree et al. (2016) [29]	UK (Europe)	Case–control	Healthy controls	74	88	29	32	NA
Dhingra et al. [30]	USA (America)	Case–control	Patients without EDS	4294^a^	4294	686	1657	NA
Wasim et al. [31]	Canada (America)	Cross‐sectional	NA	391	NA	NA	241	NA
Nee et al. [32]	USA (America)	Cross‐sectional	NA	1804^a^	NA	1661	NA	NA
Tai et al. [10]	UK (Europe)	Cross‐sectional	NA	616	NA	554	431	231
Silvernale et al. [33]	USA (America)	Cross‐sectional	NA	193	NA	131	NA	NA
Zloof et al. [34]	Israel (Middle East)	Case–control	Healthy controls	4686	1,621,721	71	NA	NA
Leganger et al. [35]	Denmark (Europe)	Case–control	Healthy controls	1319^a^	46,700	165	165	NA
Solomon et al. [16] (conference abstract)	USA (America)	Cross‐sectional	NA	75^a^	NA	39	NA	NA

^a^ Study population included multiple EDS subtypes.

### 3.2 Prevalence of Chronic Gastrointestinal Symptoms in Patients With hEDS/HSD

**FIGURE 2:**
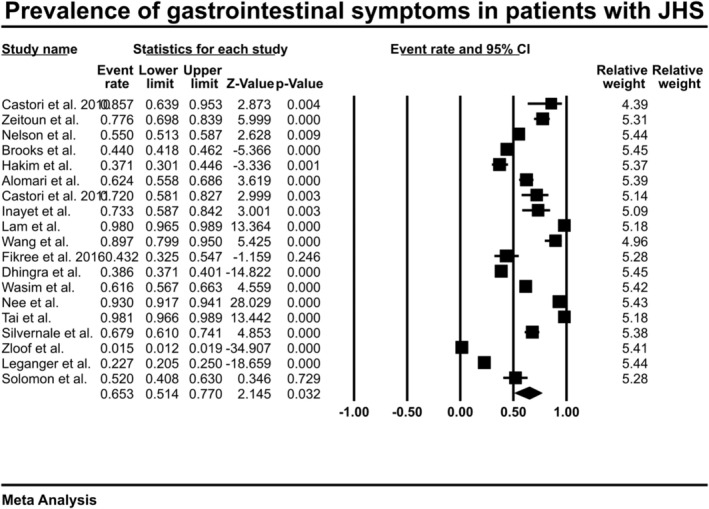
Forest plot of studies showing prevalence of chronic gastrointestinal symptoms in patients with hEDS/HSD (65.3%, 95% CI, 51.5–77.0, *I* ^2^ = 99.4, *p* = 0.0001).

Nineteen studies [9, 10, 16, 20, 21, 22, 23, 24, 25, 26, 27, 28, 29, 30, 31, 32, 33, 34, 35] with 17,455 patients with hEDS/HSD (mean age ranging from 17.2 to 44.3 years, Table S3) reported chronic gastrointestinal symptoms. Overall, 65.3% (95% CI 51.4–77.0) of patients with hEDS/HSD reported at least one chronic gastrointestinal symptom, with considerable heterogeneity in the analysis (*I* ^2^ = 99.4, *p* = 0.0001) (Figure 2). Visual inspection of the funnel plot showed possible asymmetry (Figure S1), but the results of the Egger's test (*p* = 0.16) do not confirm publication bias.

The pooled prevalence estimate includes one large study (*n* = 4686) by Zloof et al. [34], which provided an atypically low prevalence of chronic GI symptoms (1.5%), Table 1. A sensitivity analysis excluding this study demonstrated a non‐statistically significant increase in the prevalence of chronic GI symptoms in patients with hEDS/HSD (71%; 95% CI 60.1–79.9) compared to the primary analysis (65.3%, 95% CI 51.4–77.0) (Figure S47).

When stratified by geographic regions, there was no statistically significant difference between the prevalence of chronic GI symptoms in patients with hEDS/HSD in North America (65.7%, 95% CI 51.1–77.8) or Europe (76.5%, 95% CI 48.6–91.8), *p* = 0.448 (Figure S6).

### 3.3 Prevalence of Chronic Gastrointestinal Symptoms in Patients With hEDS/HSD Compared to Controls

Eight case–control studies [9, 23, 24, 27, 29, 30, 34, 35] with 13,198 patients with hEDS/HSD and 1,677,515 mixed healthy and patient controls were included in this analysis. Patients with hEDS/HSD had fourfold higher odds of having chronic gastrointestinal symptoms compared to controls (OR 4.17; 95% CI 3.02–5.77), with considerable heterogeneity noted in the analyses (*I* ^2^ = 94%, *p* < 0.001) (Figure S2). Excluding the Zloof et al. study as an outlier yields an OR of 4.52 (95% CI 3.11–6.55) compared with the primary analysis (OR 4.17, 95% CI 3.02–5.77), though the change is not statistically significant (Figure S48).

Including only four case–control studies with only healthy controls [9, 29, 34, 35], patients with hEDS/HSD had fivefold higher odds of having chronic gastrointestinal symptoms compared to controls (OR 4.62; 95% CI 2.1–10.0), with considerable heterogeneity noted in the analyses (*I* ^2^ = 96%, *p* < 0.001) (Figure S3). Including three studies with patient controls, patients with hEDS/HSD had threefold higher odds of having chronic GI symptoms compared to controls (OR 3.44; 95% = CI 2.97–4.0), with moderate heterogeneity seen in the analysis (*I* ^2^ = 58%, *p* = 0.09) (Figure S3). Overall, there was no statistically significant difference in the prevalence of GI symptoms between healthy controls and patient controls (*p* = 0.46).

### 3.4 Influence of Risk of Bias on the Prevalence of Chronic Gastrointestinal Symptoms in Patients With hEDS/HSD

*Studies with low risk of bias:* The majority (15/19, 79%) of the studies were of high quality, as assessed utilizing the JBI critical appraisal tool and NOS (Tables S4 and S5). Including only 15 high‐quality studies, the prevalence of chronic gastrointestinal symptoms in patients with hEDS/HSD was 67.3% (51.3–80.1) with considerable heterogeneity seen in the studies included in the analysis (*I* ^2^ = 99.5, *p* = 0.0001). Thus, the outcome did not change with the elimination of low‐quality studies, nor did it explain the observed heterogeneity (Figure S4, Table 2). Visual inspection of the funnel plot did not show any asymmetry (Figure S5), in keeping with the results of the Egger's test (*p* = 0.2).

**TABLE 2:** Summary of findings of the outcomes reported in this systematic review and meta‐analysis.

Symptoms	Number of studies, *n*	Number of eligible participants, *n*	Pooled prevalence, % (95% CI)	Assessment of heterogeneity between studies and publication bias
Prevalence of chronic GI symptoms in patients with hEDS/HSD	19	17,455	65.3 (51.4–77.0)	*I* ^2^ = 99.4, *p* = 0.0001
Prevalence of chronic GI symptoms in patients with hEDS/HSD (low risk of bias)	15	16,967	67.3 (51.3–80.1)	*I* ^2^ = 99.5, *p* = 0.0001
Prevalence of chronic GI symptoms in patients with hEDS/HSD (2017 international criteria)	3	677	70.6 (57.7–80.8)	*I* ^2^ = 88.2, *p* = 0.0001
Prevalence of chronic GI symptoms in patients with hEDS/HSD (pre‐2017)	16	16,778	63.7 (47.8–77.0)	*I* ^2^ = 99.5, *p* = 0.0001
Prevalence of chronic GI symptoms in patients with hEDS/HSD (outlier excluded)	18	12,769	71 (60.1–79.9)	*I* ^2^ = 99.1, *p* = 0.0001
Prevalence of heartburn in patients with hEDS/HSD	9	4249	34.7 (28.3–41.7)	*I* ^2^ = 93.5, *p* = 0.0001
Prevalence of abdominal pain in patients with hEDS/HSD	14	7721	31.3 (20.0–45.4)	*I* ^2^ = 98.5, *p* = 0.0001
Prevalence of constipation in patients with hEDS/HSD	10	6256	21.6 (12.8–34.0)	*I* ^2^ = 98.4, *p* = 0.0001
Prevalence of nausea in patients with hEDS/HSD	10	6256	21.0 (12.4–33.5)	*I* ^2^ = 98.5, *p* = 0.0001
Prevalence of dysphagia in patients with hEDS/HSD	10	6256	18.5 (10.5–30.3)	*I* ^2^ = 98.7, *p* = 0.0001
Prevalence of postprandial distress in patients with hEDS/HSD	7	5390	16.6 (7.9–31.7)	*I* ^2^ = 98.6, *p* = 0.0001
Prevalence of bloating in patients with hEDS/HSD	10	6256	11.9 (6.1–21.9)	*I* ^2^ = 97.8, *p* = 0.0001
Prevalence of vomiting in patients with hEDS/HSD	9	6122	10.7 (6.6–16.9)	*I* ^2^ = 96.7, *p* = 0.0001
Prevalence of diarrhoea in patients with hEDS/HSD	9	6122	8.2 (3.3–19.2)	*I* ^2^ = 98.4, *p* = 0.0001
Prevalence of rectal prolapse in patients with hEDS/HSD	3	2709	4.8 (1.0–19.6)	*I* ^2^ = 97.6, *p* = 0.0001
DGBI				
Prevalence of DGBI in patients with hEDS/HSD	16	16,844	44.2 (23.9–66.6)	*I* ^2^ = 99.6, *p* = 0.0001
Prevalence of functional dysphagia in patients with hEDS/HSD	4	3068	34.2 (25.7–43.8)	*I* ^2^ = 95.2, *p* = 0.0001
Prevalence of functional dyspepsia in patients with hEDS/HSD	7	5342	31.8 (19.0–48.2)	*I* ^2^ = 98.7, *p* = 0.0001
Prevalence of functional heartburn in patients with hEDS/HSD	4	3068	29.9 (23.9–36.8)	*I* ^2^ = 90.3, *p* = 0.0001
Prevalence of IBS in patients with hEDS/HSD	14	15,504	23.3 (12.9–38.3)	*I* ^2^ = 99.5, *p* = 0.0001
Prevalence of functional constipation in patients with hEDS/HSD	5	3202	19.3 (10.3–33.1)	*I* ^2^ = 97.2, *p* = 0.0001
Prevalence of faecal incontinence in patients with hEDS/HSD	3	1264	15.8 (12.4–19.8)	*I* ^2^ = 55.6, *p* = 0.105
Prevalence of belching disorders in patients with hEDS/HSD	4	3068	15.2 (8.8–25.0)	*I* ^2^ = 96.2, *p* = 0.0001
Prevalence of nausea and vomiting disorders in patients with hEDS/HSD	4	3068	14.4 (8.6–23.2)	*I* ^2^ = 95.6, *p* = 0.0001
Prevalence of rumination in patients with hEDS/HSD	4	3068	14.1 (4.9–34.2)	*I* ^2^ = 99.0, *p* = 0.0001
Prevalence of CVS in patients with hEDS/HSD	3	3023	13.4 (7.7–22.1)	*I* ^2^ = 96.2, *p* = 0.0001
Prevalence of functional bloating in patients with hEDS/HSD	4	3068	7.8 (2.8–19.9)	*I* ^2^ = 96.7, *p* = 0.0001
Prevalence of functional chest pain in patients with hEDS/HSD	4	3068	7.6 (3.0–17.7)	*I* ^2^ = 97.2, *p* = 0.0001
Prevalence of unspecified bowel disorder in patients with hEDS/HSD	4	3068	7.5 (2.2–22.6)	*I* ^2^ = 97.4, *p* = 0.0001
Prevalence of functional diarrhoea in patients with hEDS/HSD	4	3068	5.0 (1.6–14.8)	*I* ^2^ = 96.4, *p* = 0.0001
Prevalence of globus in patients with hEDS/HSD	4	3068	2.1 (1.7–2.7)	*I* ^2^ = 0.0, *p* = 0.595
Prevalence of functional biliary disorders in patients with hEDS/HSD	3	1264	1.2 (0.7–2.0)	*I* ^2^ = 0.0, *p* = 0.814
Prevalence of functional dyspepsia in a subset of patients with hEDS/HSD	4	3068	55.4 (52.4–58.3)	*I* ^2^ = 50.0, *p* = 0.112
Prevalence of IBS in a subset of patients with hEDS/HSD	4	3068	54.0 (49.5–58.4)	*I* ^2^ = 76.1, *p* = 0.006
Extraintestinal comorbidities				
Prevalence of chronic fatigue in patients with hEDS/HSD	5	1442	49.0 (34.6–63.6)	*I* ^2^ = 93.9, *p* = 0.0001
Prevalence of GERD in patients with hEDS/HSD	5	2370	41.3 (27.2–57.0)	*I* ^2^ = 94.0, *p* = 0.0001
Prevalence of migraine in patients with hEDS/HSD	3	776	38.2 (19.9–60.5)	*I* ^2^ = 88.3, *p* = 0.0001
Prevalence of OI in patients with hEDS/HSD	7	3816	35.9 (26.6–46.4)	*I* ^2^ = 96.4, *p* = 0.0001
Prevalence of POTS in patients with hEDS/HSD	3	1264	21.9 (5.2–59.2)	*I* ^2^ = 99.5, *p* = 0.0001
Prevalence of fibromyalgia in patients with hEDS/HSD	6	1713	27.9 (16.0–44.0)	*I* ^2^ = 96.3, *p* = 0.0001
**Odds ratio**	**Number of studies, *n***	**Number of cases, *n* (Number of controls, *n*)**	**Odds ratio (95% CI)**	**Assessment of heterogeneity between studies and publication bias**
Odds ratio of chronic GI symptom prevalence in patients with hEDS/HSD vs. mixed controls	8	13,198 (1,677,515)	4.17 (3.0–5.8)	*I* ^2^ = 94.1, *p* = 0.0001
Odds ratio of chronic GI symptom prevalence in patients with hEDS/HSD vs. healthy controls	5	6852 (1,669,162)	4.62 (2.1–10.0)	*I* ^2^ = 96.1, *p* = 0.0001
Odds ratio of chronic GI symptom prevalence in patients with hEDS/HSD vs. patient controls	3	6346 (8353)	3.44 (3.0–4.0)	*I* ^2^ = 58.1, *p* = 0.09
Odds ratio of DGBI prevalence in patients with hEDS/HSD vs. mixed controls	7	13,028 (1,677,465)	4.97 (3.1–8.0)	*I* ^2^ = 94.9, *p* = 0.0001

### 3.5 Influence of the 2017 International Diagnostic Criteria for EDS/HSD on the Prevalence of Chronic Gastrointestinal Symptoms in Patients With hEDS/HSD

We also conducted a subgroup analysis comparing the prevalence of chronic GI symptoms in patients with hEDS/HSD in studies conducted before and after 2017. Sixteen studies included in this meta‐analysis were conducted pre 2017 and 3 post 2017. There was no statistically significant difference in the prevalence of GI symptoms in patients with hEDS/HSD based on studies conducted pre 2017 (70.6%; 95% CI 57.7–80.8) compared to those conducted post 2017 (63.7%; 95% CI 47.8–77.0) (Figure S46).

### 3.6 Most Prevalent Individual Gastrointestinal Symptoms in Patients With hEDS/HSD

Heartburn was the most prevalent gastrointestinal symptom reported in patients with hEDS/HSD (34.7%, 95% CI 28.3–41.7, nine studies [9, 10, 21, 22, 25, 27, 28, 29, 32]), followed by abdominal pain (31.3%, 95% CI 20.0–45.4, 14 studies [9, 10, 16, 20, 21, 22, 23, 25, 26, 27, 28, 29, 32, 35]), constipation (21.6%, 95% CI 12.8–34.0, 10 studies [9, 10, 21, 22, 23, 25, 27, 28, 29, 32]), nausea (21%, 95% CI 12.4–33.5, 10 studies [9, 10, 21, 22, 23, 25, 27, 28, 29, 32]), dysphagia (18.5%, 95% CI 10.5–30.3, 10 studies [9, 10, 21, 22, 23, 25, 27, 28, 29, 32]), postprandial distress (16.6%, 95% CI 7.9–31.7, seven studies [10, 21, 22, 23, 28, 29, 32]), bloating (11.9%, 95% CI 6.1–21.9, 10 studies [9, 10, 21, 22, 23, 25, 27, 28, 29, 32]), vomiting (10.7%, 95% CI 6.6–16.9, nine studies [9, 10, 22, 23, 25, 27, 28, 29, 32]), diarrhoea (8.2%, 95% CI 3.3–19.2, nine studies [9, 10, 22, 23, 25, 27, 28, 29, 32]) and rectal prolapse (4.8%, 95% CI 1.0–19.6, three studies [22, 25, 32]). There was substantial heterogeneity seen in these analyses (Table 2) (Figures S7–S21).

### 3.7 Prevalence of Gastrointestinal Disorders Including DGBI in Patients With hEDS/HSD

GERD was the most common gastrointestinal condition reported in 41.3% (95% CI 27.2–57.0, five studies [20, 23, 25, 26, 29]) of hEDS/HSD patients, with considerable heterogeneity (*I* ^2^ = 94.0, *p* = 0.0001) (Figure S22).

#### 3.7.1 Prevalence of DGBI in Patients With hEDS/HSD

**FIGURE 3:**
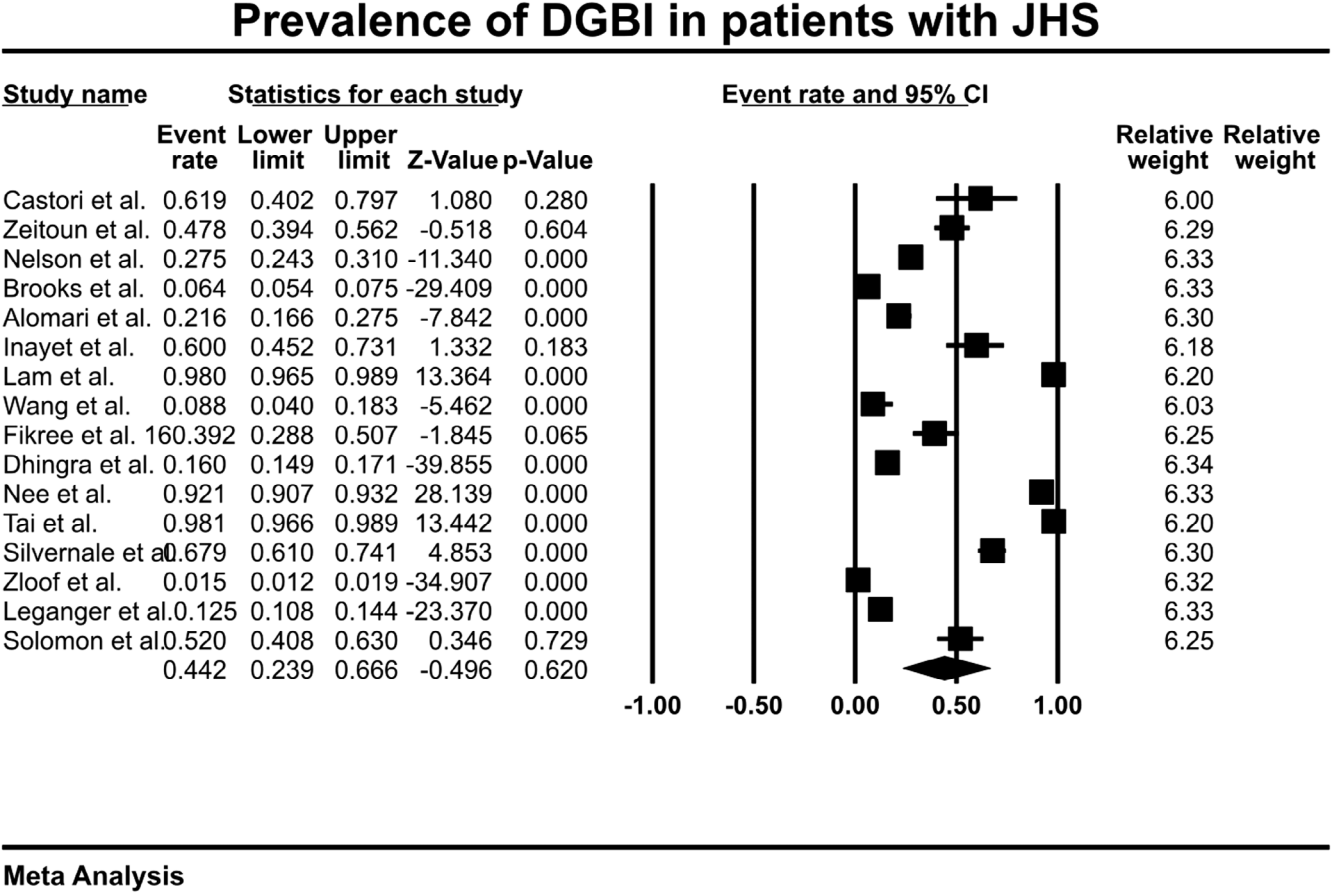
Forest plot of studies showing prevalence of DGBI in patients with hEDS/HSD (44.2%, 95% CI, 23.9–66.6, *I* ^2^ = 99.6, *p* = 0.0001).

Overall, 16 [9, 10, 16, 20, 21, 22, 23, 25, 27, 28, 29, 30, 32, 33, 34, 35] studies with 16,844 patients with hEDS/HSD included in this meta‐analysis reported the prevalence of DGBI in hEDS/HSD. An overlapping DGBI was prevalent in 44.2% (95% CI, 23.9–66.6, Figure 3) of patients with hEDS/HSD, with substantial heterogeneity in the analysis (*I* ^2^ = 99.6, *p* = 0.0001). Visual inspection of the funnel plot did not show any asymmetry (Figure S23), in keeping with the results of the Egger's test (*p* = 0.2).

Functional dysphagia was the most prevalent DGBI reported in 34.2% (95% CI 25.7–43.8, four studies [9, 10, 27, 32]) of patients with hEDS/HSD, followed by functional dyspepsia (31.8%, 95% CI 19.0–48.2, seven studies [9, 10, 23, 27, 32, 33, 36]), functional heartburn (29.9%, 95% CI 23.9–36.8, four studies [9, 10, 27, 32]), IBS (23.3%, 95% CI 12.9–38.3, 14 studies [9, 10, 16, 21, 22, 23, 25, 27, 28, 29, 30, 32, 33, 34]), functional constipation (19.3%, 95% CI 10.3–33.1, five studies [9, 10, 21, 27, 32]), faecal incontinence (15.8%, 95% CI 12.4–19.8, three studies [9, 10, 27]), belching disorders (15.2%, 95% CI 8.8–25.0, four studies [9, 10, 27, 32]), nausea and vomiting disorders (14.4%, 95% CI 8.6–23.2, four studies [9, 10, 27, 32]), rumination (14.1%, 95% CI 8.6–23.2, four studies [9, 10, 27, 32]), cyclical vomiting syndrome (CVS) (13.4%, 95% CI 7.7–22.1, three studies [9, 10, 27]), functional bloating (7.8%, 95% CI 2.8–19.9, four studies [9, 10, 27, 32]), functional chest pain (7.6%, 95% CI 3.0–17.7, four studies [9, 10, 27, 32]), unspecified bowel disorder (7.5%, 95% CI 2.2–22.6, four studies [9, 10, 23, 27]), functional diarrhoea (5%, 95% CI 1.6–14.8, four studies [9, 10, 27, 32]), globus (2.1%, 95% CI 1.7–2.7, four studies [9, 10, 27, 32]) and functional biliary disorders (1.2%, 95% CI 0.7–0, three studies [9, 10, 27]). With the exception of functional biliary disorders and globus, which demonstrated low heterogeneity, all other analyses demonstrated considerable heterogeneity (Table 2, Figures S24–S40).

However, only four studies [9, 10, 27, 32] with 3068 patients with hEDS/HSD included in this meta‐analysis reported the prevalence of 12 different DGBI within the same population, allowing for comparative analysis. Interestingly, in this subgroup analyses, functional dyspepsia was the most prevalent DGBI, reported in 55.4% (95% CI 52.5–58.3) of patients with hEDS/HSD, followed by IBS (54.0%, 95% CI 49.5–58.4), functional dysphagia (34.2%, 95% CI 25.7–43.8), functional heartburn (29.9%, 95% CI 23.9–36.8), functional constipation (19.3%, 95% CI 10.3–33.1), belching disorders (15.2%, 95% CI 8.8–25.0), nausea and vomiting disorders (14.4%, 95% CI 8.6–23.2), rumination (14.1%, 95% CI 8.6–23.2), functional bloating (7.8%, 95% CI 2.8–19.9), functional chest pain (7.6%, 95% CI 3.0–17.7), functional diarrhoea (5%, 95% CI 1.6–14.8) and globus (2.1%, 95% CI 1.7–2.7).

#### 3.7.2 Prevalence of DGBI in Patients With hEDS/HSD Compared to Controls

**FIGURE 4:**
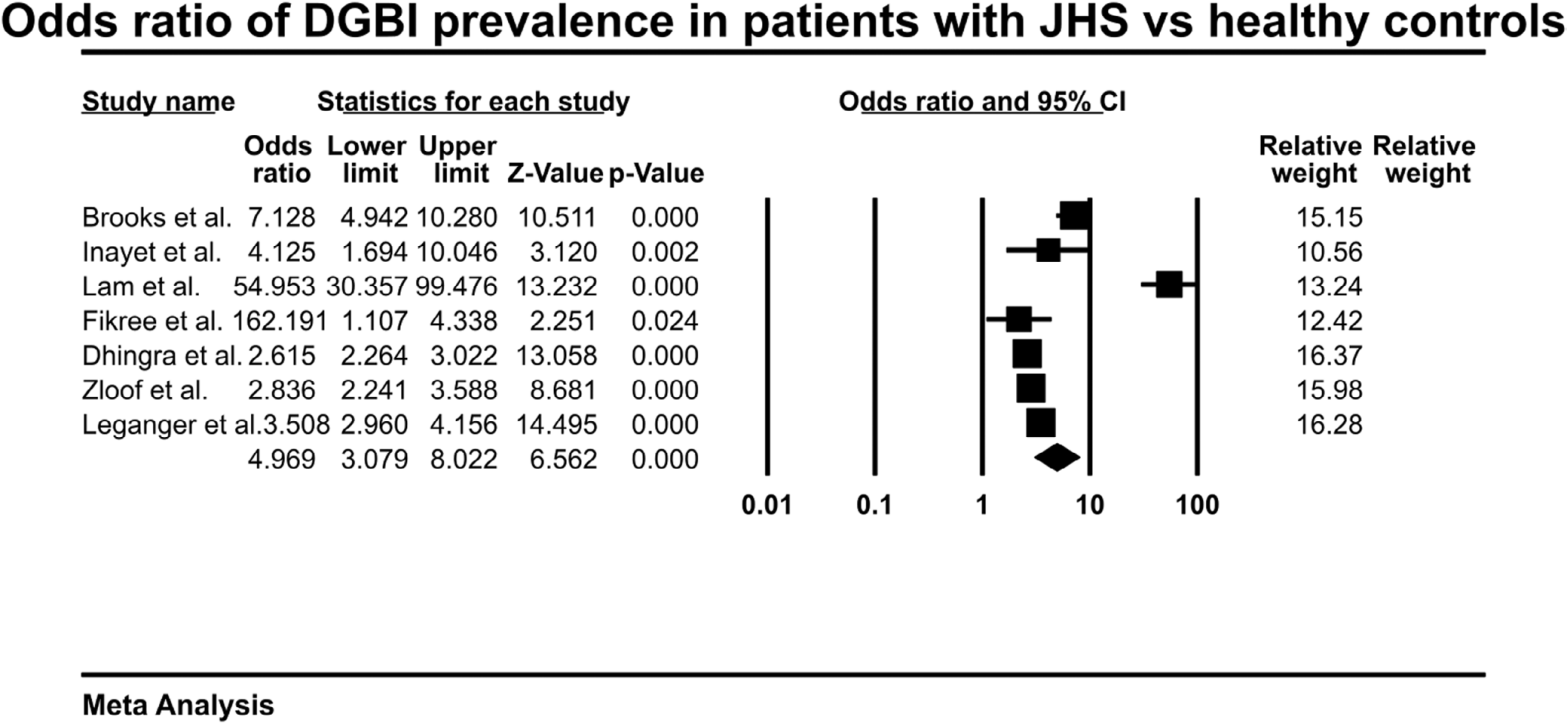
Forest plot of studies showing odds ratio of DGBI prevalence in patients with hEDS/HSD vs. mixed controls (OR 4.969, 95% CI 3.079–8.022, *I* ^2^ = 94.9, *p* = 0.0001).

Seven case–control studies [9, 23, 27, 29, 30, 34, 35] with 13,028 patients with hEDS/HSD and 1,677,465 controls were included in this analysis. Patients with hEDS/HSD had nearly fivefold higher odds of having an overlapping DGBI compared to controls (OR 4.97; 95% CI 3.08–8.02), with considerable heterogeneity noted in the analyses (*I* ^2^ = 94.9%, *p* < 0.001) (Figure 4).

### 3.8 Prevalence of Extraintestinal Conditions in Patients With hEDS/HSD

Chronic fatigue was the most common extraintestinal symptom seen in patients with hEDS/HSD (49%, 95% CI 34.6–63.6, five studies), followed by migraine (38.2%, 95% CI 19.9–60.5, three studies), and fibromyalgia (27.9%, 95% CI 16.0–44.0, six studies). There was substantial heterogeneity seen in these analyses (Table 2, Figures S41–S43).

Finally, we found that orthostatic intolerance was prevalent in 35.9% (95% CI 26.6–46.4, seven studies [10, 22, 23, 24, 25, 26, 28]) of patients with hEDS/HSD, with considerable heterogeneity in the analysis (*I* ^2^ = 96.4, *p* = 0.0001) (Figure S44). The prevalence of Postural Orthostatic Tachycardia Syndrome (POTS) in patients with hEDS/HSD was 21.9% (95% CI 5.2–59.1, three studies [10, 23, 25]), with considerable heterogeneity in the analysis (*I* ^2^ = 99.5, *p* = 0.0001) (Figure S45).

## 4 Discussion

To the best of our knowledge, this is the first systematic review and meta‐analysis including 19 studies to assess the prevalence of GI symptoms in 17,455 patients with hEDS/HSD. Overall, two‐thirds of patients with pre‐existing hEDS/HSD reported having chronic GI symptoms, with heartburn and abdominal pain identified as the most frequently reported complaints. Importantly, patients with hEDS/HSD exhibited a fourfold increase in odds of experiencing chronic GI symptoms compared to controls. Additionally, 44.2% of patients with hEDS/HSD were diagnosed with a DGBI, with functional dysphagia being the most common DGBI with a prevalence of 34.2%. Chronic fatigue was the most common extraintestinal condition reported by almost 50% of patients with hEDS/HDS followed by migraine and fibromyalgia. Notably, orthostatic intolerance affected over a third of the patients with hEDS/HSD, with 21.9% of patients with hEDS/HSD being diagnosed with POTS.

We noted considerable heterogeneity across the studies included in the primary and subgroup analyses. This heterogeneity is evident not only from the *I* ^2^ statistics, but also from the wide range of prevalence estimates observed between studies in the Forest plots. Consequently, pooled estimates were interpreted with caution, rather than taken at face value. Based on the findings presented here, we believe several factors contribute to the clinical heterogeneity: first, 15/19 studies employed a retrospective study design, limiting the ability to allow for the determination of temporality and causality. Additionally, 11/19 were conducted in secondary and tertiary care institutions, which may underrepresent mild cases of hEDS/HSD. Furthermore, most studies (12/19) did not include a control group, limiting the generalizability and comparative strength of their findings. Four studies relied on questionnaires, introducing recall bias, which we attempted to explore the potential effects of by reporting the pooled prevalence values of self‐reported chronic GI symptoms and clinically diagnosed DGBI separately to avoid misinterpretation. Eight studies did not discern between EDS subtypes, which may have led to an overestimation of hEDS prevalence. After performing the literature search, only two studies including paediatric patients fulfilled inclusion criteria; hence, we were unable to conduct a subgroup analysis. One study [16] recruited a highly selective population of paediatric patients with POTS and EDS, limiting generalizability to the broader hEDS/HSD population.

The Zloof et al. [34] study appeared as an outlier with an extremely low prevalence of chronic GI symptoms in patients with hEDS/HSD, despite having the largest population. Its results are likely driven by its scale and the long period analysed (1998–2020) that covered the entire adolescent population of Israel, unlike the other studies that used community samples. However, it is worth noting that the Zloof et al. study has notable gaps in missing data handling, which might have led to an underestimation of the chronic GI symptom prevalence. A sensitivity analysis excluding the Zloof et al. showed no statistically significant change in the pooled prevalence of chronic GI symptoms in patients with hEDS/HSD. Finally, co‐morbidities were often self‐reported rather than confirmed by a physician.

Notably, one of the important criteria contributing to clinical heterogeneity was the use of varying diagnostic criteria and standards for EDS and HSD across studies. 16/19 studies included in this meta‐analysis were conducted before 2017, when the new diagnostic criteria for hEDS were published. Consequently, the majority have used the Brighton Criteria, which was the standard method for diagnosing joint hypermobility syndrome (JHS). However, these diagnostic criteria are based on expert opinion, which is changing over time. It is generally believed that patients diagnosed with JHS would meet the 2017 criteria for hEDS or fall under the umbrella of the HSD, where individuals satisfy many but not necessarily all hEDS criteria, although this relationship has not been definitively studied. Therefore, further research is needed in patients with hEDS/HSD using the current 2017 hEDS criteria to determine the overlap with DGBI. The differences in the diagnostic criteria, which are evolving based on expert consensus, can certainly affect the prevalence estimates and introduce misclassification bias. Moreover, the overlap in presentation between hEDS and HSD is substantial; therefore, it is appropriate to consider them together until biomarker studies can differentiate between the two. However, we did not find a statistically significant difference in the prevalence of chronic GI symptoms in patients with hEDS/HSD in studies conducted before and after 2017.

We conducted a sensitivity analysis including only high‐quality studies, but this did not explain the high heterogeneity or the risk of publication bias. Thus, the substantial to considerable between‐study heterogeneity and high risk of bias could at least partially be explained by the inherent limitations and specifics of the studies included in this meta‐analysis. This would suggest that the prevalence of chronic gastrointestinal symptoms in hEDS/HSD could have been overestimated. Other limitations of this systematic review and meta‐analysis include the small sample size of some studies, two of which had < 50 participants, introducing sampling bias and some subgroup analyses are based on a small number of studies and the heterogeneity of the diagnostic methodology. Thus, the findings of this systematic review and meta‐analysis need to be interpreted with caution.

Gastrointestinal symptoms are highly prevalent in patients with hEDS/HSD, with prevalence rates for at least one gastrointestinal symptom ranging from 30% to 96% [37], and these rates then increase with age [37]. Considering their frequency and the related impact on quality of life, the association between gastrointestinal involvement and hEDS/HSD suggests clinical importance. In the current meta‐analysis, at least two‐thirds of patients with hEDS/HSD reported at least one chronic gastrointestinal symptom, which was five times more prevalent than in healthy controls. Notably, heartburn was the most common symptom, affecting one‐third of patients, followed by abdominal pain. Additionally, approximately 20% reported experiencing constipation, nausea, dysphagia, and postprandial distress.

hEDS/HSD may be associated with altered gastrointestinal innervation as reflected by, for example, gastrointestinal dysmotility; however, the existing evidence is primarily drawn from retrospective studies at tertiary centres, leaving the true prevalence uncertain [37]. Unfortunately, we were unable to extract sufficient data to conduct subgroup analyses in regard to motility changes in patients with hEDS/HSD.

In this meta‐analysis, we found that 44% of patients with hEDS/HSD reported a DGBI. Furthermore, DGBI were sixfold more prevalent in patients with hEDS/HSD compared to healthy controls. Despite functional dyspepsia and functional constipation being the most common DGBI within the general population [38, 39], the most prevalent DGBI in patients with hEDS/HSD was functional dysphagia, affecting approximately 33% of patients, followed closely by functional dyspepsia and functional heartburn. Other common DGBIs reported by approximately 20% of patients with hEDS/HSD included IBS and functional constipation, with around 15% of patients reporting faecal incontinence, belching disorders, nausea and vomiting disorders, and cyclical vomiting syndrome. However, this likely reflects small‐sample bias, since pooled estimates for most DGBIs (except functional dyspepsia and IBS) derive from four studies, while FD and IBS include 7 and 14 studies, respectively. When we conducted subgroup analysis including only four studies reporting majority of DGBI in hEDS/HSD, functional dyspepsia followed by IBS were the most prevalent DGBI, at 55% and 54% respectively. Our findings are aligned with those reported in the literature, with DGBI frequently reported in patients with hEDS/HSD [2, 9, 10]. A recent study found increased prevalence of bowel, gastroduodenal and oesophageal functional disorders in hEDS/HSD when compared with age‐matched controls [9]. Notably, they reported a higher prevalence of patients with HSD/hEDS who had 2 or more DGBI compared to the population controls (84% vs. 15%, *p* < 0.001) [9].

Chronic gastrointestinal symptoms and DGBI in these patients are highly prevalent and often severe [24], leading to polypharmacy that carries risks of drug–drug interactions and adverse effects [30]. Nutritional compromises may require invasive interventions [25], such as enteral and parenteral nutrition, resulting in increased healthcare utilization and significantly impacting the quality of life for patients with hEDS/HSD [21, 27, 29].

Patients with hEDS/HSD also have a high burden of extraintestinal conditions [2]. Almost 50% of patients with hEDS/HSD had chronic fatigue, aligning with previously published findings [2], followed by a high burden of migraines and fibromyalgia, also commonly reported in literature [2]. In the current meta‐analysis, we found that one‐third of patients with hEDS/HSD have orthostatic intolerance, while nearly 20% were diagnosed with POTS. There is a growing body of literature [40, 41, 42, 43, 44, 45], on the overlap or co‐existence of orthostatic intolerance, specifically POTS, hEDS/HSD, MCAS and chronic gastrointestinal symptoms, or DGBI. Although several studies have reported an association among the three conditions, the precise mechanism underlying their overlap has yet to be fully explored using contemporary diagnostic criteria for these conditions [42]. Several other extraintestinal conditions have been reported in patients with hEDS/HSD in the studies included in this meta‐analysis including MCAS [23, 25, 30], anatomical abnormalities (e.g., diverticulosis, rectoceles, rectal prolapse) [22], eating disorders [46], mental health co‐morbidities (e.g., anxiety, depression, low mood) [31] but we were unable to conduct subgroup analyses due to limited no of studies reporting these data.

Three previous literature reviews examining the gastrointestinal involvement in hEDS/HSD have been published in 2015 [47] (12 studies), 2016 [11] (11 studies) and 2017 [36] (21 studies), exploring the pathophysiology, presentations and overlapping conditions. Importantly, a clinical practice update expert review has been published recently [41], stressing the importance of awareness of the potential for overlap of hEDS or HSDs, POTS and/or MCAS with their gastrointestinal manifestations, emphasizing the necessity for a multidisciplinary management approach. The strength of the current study is that we not only assessed the available literature systematically but also performed a meta‐analysis. We also systematically explored potential explanations for the reasons for the high heterogeneity and high risk of bias observed in our analyses, outlined above.

In summary, the findings of this systematic review and meta‐analysis suggest that 65% of patients with hEDS/HSD experience concurrent gastrointestinal symptoms, with heartburn being the most common symptom reported by 35% of patients. Other common concurrent gastrointestinal symptoms include abdominal pain, constipation, nausea, dysphagia, postprandial distress, bloating, vomiting, faecal incontinence, and diarrhoea. Although multifactorial, the pathophysiology of gastrointestinal symptoms in hEDS/HSD remains unknown; their management remains challenging, resulting in increased healthcare utilization [13].

Patients with hEDS/HSD experience a considerable burden of DGBI and other chronic extraintestinal comorbidities, often exceeding DGBI prevalence. Nearly 50% report chronic fatigue as the most common comorbidity, followed by migraine and fibromyalgia. Approximately one‐third of patients report DGBI, with functional dysphagia being the most prevalent DGBI. Additionally, OI is reported in one‐third of patients, with the prevalence of POTS at 21.9%.

While the results of this systematic review and meta‐analysis are consistent with an association between hEDS/HSD and GI symptoms or DGBI, the precise mechanisms for these associations need to be defined before causality can be assumed. Timely recognition of chronic gastrointestinal symptoms and other co‐morbidities in hEDS/HSD as a pathophysiological cause of symptoms and adequate integrated multidisciplinary treatment are necessary to improve patient quality of life, reduce healthcare utilization, and achieve positive long‐term outcomes. However, given substantial heterogeneity across analyses, the results should be interpreted with caution.

## Author Contributions

**Gerald Holtmann:** conceptualization, investigation, writing – original draft, writing – review and editing, methodology, formal analysis, data curation. **Michael P. Jones:** validation, supervision. **Samuel Nurko:** validation, supervision. **Qasim Aziz:** validation, supervision. **Laurie Keefer:** validation, supervision. **Dmitrii Kulin:** conceptualization, investigation, writing – original draft, methodology, writing – review and editing, formal analysis, data curation. **Kyle Staller:** validation, supervision. **Douglas A. Drossman:** validation, supervision. **Thomas Fairlie:** conceptualization, investigation, writing – original draft, methodology, writing – review and editing, formal analysis, data curation. **Ayesha Shah:** conceptualization, investigation, writing – original draft, methodology, writing – review and editing, formal analysis, data curation.

## Funding

This work was supported by the National Health and Medical Research Council (APP2004495) and NHMRC CRE in Transforming Gut Health (APP2035319).

## Conflicts of Interest

G.H. reports to be on the advisory boards of Australian Biotherapeutics, Glutagen, Bayer and received research support from Bayer. He serves on the Boards of the West Moreton Hospital and Health Service (WMHHS), Queensland, UQ Healthcare, Brisbane and is Vice President of Gastro‐Liga, Germany and is Chair of the WMHHS Board Quality and Safety Committee. He serves as Editor of the Gastro‐Liga Newsletter. He is on the Research Committee of the Royal Australasian College of Physicians. G.H. acknowledges funding from the National Health and Medical Research Council (NHMRC) for the Centre for Research Excellence in Digestive Health. G.H. holds an MRFF and an NHMRC Ideas grant. A.S. acknowledges funding from the National Health and Medical Research Council (NHMRC) for the Centre for Research Excellence in Transforming Gut Health. A.S. holds an MRFF, NHMRC Ideas grant and an NHMRC Emerging Leadership Fellow (EL1). K.S. reports serving as a consultant to AbbVie Ardelyx, Atmo, Ferring, Gemelli Biotech, Laborie, MindSet Health, Salix, and Takeda. He has received research support from Ardelyx. L.K. acknowledges research funds from Ardelyx and the Leona M and Harold B Helmsley Charitable Trust, and serves as a consultant for AbbVie, Pfizer, Eli Lilly, and J&J, and is an equity owner/co‐founder/advisor for Trellus Health. S.N. acknowledges research funds from Ardelyx and serves as a consultant for AbbVie and Ardelyx. Q.A. serves on the medical advisory board for Ehlers Danlos Society, USA and has received a peer‐reviewed research grant from this society and the Ehlers Danlos Support Group UK. He has had research funding from Takeda pharmaceuticals, Dr. Falk, and Classado pharmaceuticals.

## Supporting information


**Figure S1:** Funnel plot of studies showing prevalence rates of Chronic GI symptoms in patients with hEDS/HSD.
**Figure S2:** Forest plot of studies showing odds ratio of the prevalence rates of chronic GI symptoms in patients with hEDS/HSD vs. mixed controls (OR 4.29, 95% CI 3.05–6.03, *I* ^2^ = 95%, *p* < 0.001).
**Figure S3:** Forest plot of studies showing odds ratio of the prevalence rates of chronic GI symptoms in patients with hEDS/HSD, stratified by control group.
**Figure S4:** Forest plot of studies with low risk of bias showing prevalence rates of chronic GI symptoms in patients with hEDS/HSD (PP 67.3%, 95% CI, 51.3–80.1, *I* ^2^ = 99.521, *p* = 0.0001).
**Figure S5:** Funnel plot of studies with low risk of bias showing prevalence rates of chronic GI symptoms in patients with hEDS/HSD.
**Figure S6:** Forest plot of studies showing prevalence rates of chronic GI symptoms in patients with hEDS/HSD, stratified by geographic region.
**Figure S7:** Forest plot of studies showing prevalence rates of heartburn in patients with hEDS/HSD (PP 34.7%, 95% CI 28.3–41.7, *I* ^2^ = 93.501, *p* = 0.0001).
**Figure S8:** Forest plot of studies showing prevalence rates of abdominal pain in patients with hEDS/HSD (PP 31.3%, 95% CI 20.0–45.4, *I* ^2^ = 98.521, *p* = 0.0001).
**Figure S9:** Funnel plot of studies showing prevalence rates of abdominal pain in patients with hEDS/HSD.
**Figure S10:** Forest plot of studies showing prevalence rates of constipation in patients with hEDS/HSD (PP 21.6%, 95% CI 12.8–34.0, *I* ^2^ = 98.393, *p* = 0.0001).
**Figure S11:** Funnel plot of studies showing prevalence rates of constipation in patients with hEDS/HSD.
**Figure S12:** Forest plot of studies showing prevalence rates of nausea in patients with hEDS/HSD (PP 21.0%, 95% CI 12.4–33.5, *I* ^2^ = 98.521, *p* = 0.0001).
**Figure S13:** Funnel plot of studies showing prevalence rates of nausea in patients with hEDS/HSD.
**Figure S14:** Forest plot of studies showing prevalence rates of dysphagia in patients with hEDS/HSD (PP 18.5%, 95% CI 10.5–30.3, *I* ^2^ = 98.653, *p* = 0.0001).
**Figure S15:** Funnel plot of studies showing prevalence rates of dysphagia in patients with hEDS/HSD.
**Figure S16:** Forest plot of studies showing prevalence rates of postprandial distress in patients with hEDS/HSD (PP 16.6%, 95% CI 7.9–31.7, *I* ^2^ = 98.629, *p* = 0.0001).
**Figure S17:** Forest plot of studies showing prevalence rates of bloating in patients with hEDS/HSD (PP 11.9%, 95% CI 6.1–21.9, *I* ^2^ = 97.849, *p* = 0.0001).
**Figure S18:** Funnel plot of studies showing prevalence rates of bloating in patients with hEDS/HSD.
**Figure S19:** Forest plot of studies showing prevalence rates of vomiting in patients with hEDS/HSD (PP 10.7%, 95% CI 6.6–16.9, *I* ^2^ = 96.716, *p* = 0.0001).
**Figure S20:** Forest plot of studies showing prevalence rates of diarrhoea in patients with hEDS/HSD (PP 8.2%, 95% CI 3.3–19.2, *I* ^2^ = 98.423, *p* = 0.0001).
**Figure S21:** Forest plot of studies showing prevalence rates of rectal prolapse in patients with hEDS/HSD (PP 4.8%, 95% CI 1.0–19.6, *I* ^2^ = 97.577, *p* = 0.0001).
**Figure S22:** Forest plot of studies showing prevalence rates of GERD in patients with hEDS/HSD (PP 41.3%, 95% CI 27.2–57.0, *I* ^2^ = 94.009, *p* = 0.0001).
**Figure S23:** Funnel plot of studies showing prevalence rates of DGBI in patients with hEDS/HSD.
**Figure S24:** Forest plot of studies showing prevalence rates of functional dysphagia in patients with hEDS/HSD (PP 34.2%, 95% CI 25.7–43.8, *I* ^2^ = 95.159, *p* = 0.0001).
**Figure S25:** Forest plot of studies showing prevalence rates of functional dyspepsia in patients with hEDS/HSD (PP 31.8%, 95% CI 19.0–48.2, *I* ^2^ = 98.683, *p* = 0.0001).
**Figure S26:** Forest plot of studies showing prevalence rates of functional heartburn in patients with hEDS/HSD (PP 29.9%, 95% CI 23.9–36.8, *I* ^2^ = 90.301, *p* = 0.0001).
**Figure S27:** Forest plot of studies showing prevalence rates of IBS in patients with hEDS/HSD (PP 23.3%, 95% CI 12.9–38.3, *I* ^2^ = 99.507, *p* = 0.0001).
**Figure S28:** Funnel plot of studies showing prevalence rates of IBS in patients with hEDS/HSD.
**Figure S29:** Forest plot of studies showing prevalence rates of functional constipation in patients with hEDS/HSD (PP 19.3%, 95% CI 10.3–33.1, *I* ^2^ = 97.232, *p* = 0.0001).
**Figure S30:** Forest plot of studies showing prevalence rates of faecal incontinence in patients with hEDS/HSD (PP 15.8%, 95% CI 12.4–19.8, *I* ^2^ = 55.563, *p* = 0.105).
**Figure S31:** Forest plot of studies showing prevalence rates of belching disorders in patients with hEDS/HSD (PP 15.2%, 95% CI 8.8–25.0, *I* ^2^ = 96.162, *p* = 0.0001).
**Figure S32:** Forest plot of studies showing prevalence rates of nausea and vomiting disorders in patients with hEDS/HSD (PP 14.4%, 95% CI 8.6–23.2, *I* ^2^ = 95.602, *p* = 0.0001).
**Figure S33:** Forest plot of studies showing prevalence rates of rumination in patients with hEDS/HSD (PP 14.1%, 95% CI 4.9–34.2, *I* ^2^ = 98.995, *p* = 0.0001).
**Figure S34:** Forest plot of studies showing prevalence rates of CVS in patients with hEDS/HSD (PP 13.4%, 95% CI 7.7–22.1, *I* ^2^ = 96.166, *p* = 0.0001).
**Figure S35:** Forest plot of studies showing prevalence rates of functional bloating in patients with hEDS/HSD (PP 7.8%, 95% CI 2.8–19.9, *I* ^2^ = 96.668, *p* = 0.0001).
**Figure S36:** Forest plot of studies showing prevalence rates of functional chest pain in patients with hEDS/HSD (PP 7.6%, 95% CI 3.0–17.7, *I* ^2^ = 97.238, *p* = 0.0001).
**Figure S37:** Forest plot of studies showing prevalence rates of unspecified bowel disorder in patients with hEDS/HSD (PP 7.5%, 95% CI 2.2–22.6, *I* ^2^ = 97.397, *p* = 0.0001).
**Figure S38:** Forest plot of studies showing prevalence rates of functional diarrhoea in patients with hEDS/HSD (PP 5.0%, 95% CI 1.6–14.8, *I* ^2^ = 96.419, *p* = 0.0001).
**Figure S39:** Forest plot of studies showing prevalence rates of globus in patients with hEDS/HSD (PP 2.1%, 95% CI 1.7–2.7, *I* ^2^ = 0.0001, *p* = 0.595).
**Figure S40:** Forest plot of studies showing prevalence rates of functional biliary disorders in patients with hEDS/HSD (PP 1.2%, 95% CI 0.7–2.0, *I* ^2^ = 0.0001, *p* = 0.814).
**Figure S41:** Forest plot of studies showing prevalence rates of chronic fatigue in patients with hEDS/HSD (PP 49%, 95% CI 34.6–63.6, *I* ^2^ = 93.899, *p* = 0.0001).
**Figure S42:** Forest plot of studies showing prevalence rates of migraine in patients with hEDS/HSD (PP 38.2%, 95% CI 19.9–60.5, *I* ^2^ = 88.309, *p* = 0.0001).
**Figure S43:** Forest plot of studies showing prevalence rates of fibromyalgia in patients with hEDS/HSD (PP 27.9%, 95% CI 16.0–44.0, *I* ^2^ = 96.34, *p* = 0.0001).
**Figure S44:** Forest plot of studies showing prevalence rates of orthostatic intolerance in patients with hEDS/HSD (PP 35.9%, 95% CI 26.6–46.4, *I* ^2^ = 96.397, *p* = 0.0001).
**Figure S45:** Forest plot of studies showing prevalence rates of POTS in patients with hEDS/HSD (PP 21.9%, 95% CI 5.2–59.1, *I* ^2^ = 99.479, *p* = 0.0001).
**Figure S46:** Forest plot of studies showing prevalence of chronic GI symptoms ion patients with hEDS/HSD, stratified by diagnostic criteria.
**Figure S47:** Forest plot of studies showing prevalence rates of chronic GI symptoms in patients with hEDS/HSD with an excluded outlier (PP 71.0%, 95% CI, 60.1–79.9, *I* ^2^ = 99.091, *p* = 0.0001).
**Figure S48:** Forest plot of studies showing odds ratio of the prevalence rates of chronic GI symptoms in patients with hEDS/HSD vs. mixed controls with an excluded outlier (OR 4.52, 95% CI 3.11–6.55, *I* ^2^ = 95%, *p* < 0.001).
**Table S1:** PubMed search strategy from University of Queensland librarian.
**Table S2:** Studies excluded from the systematic review and meta‐analysis with reasoning.
**Table S3:** Subject characteristics, inclusion and exclusion criteria for the studies included in the systematic review and meta‐analysis.
**Table S4:** Joanna Briggs Institute critical appraisal for assessment of quality of studies included in this systematic review and meta‐analysis.
**Table S5:** Newcastle‐Ottawa scale for assessment of case–control studies included in this systematic review and meta‐analysis.
**Table S6:** Hartung‐Knapp adjustment calculated values.

**File S1:** PRISMA checklist.

## Data Availability

Aggregate, rather than individual‐level, data were included in these analyses from published manuscripts and conference publications, which are publicly available.
