# Supplementary material for: Meta‐Analysis: Chronic Gastrointestinal Symptoms and Comorbidities in Hypermobile Ehlers–Danlos Syndrome and Hypermobility Spectrum Disorders
Source: Aliment Pharmacol Ther. 2026 Jul 12;64(5):574–89. doi: 10.1111/apt.70856 (PMC13466160; doi:10.1111/apt.70856)
Supplement: Supplementary file 1 [file APT-64-574-s001.docx]

**Supplementary material prepared for:**

**Legend of Figures:**

**Figure S1:** Funnel plot of studies showing prevalence rates of Chronic GI symptoms in patients with hEDS/HSD

**Figure S2:** Forest plot of studies showing odds ratio of the prevalence rates of chronic GI symptoms in patients with hEDS/HSD vs mixed controls (OR 4.29, 95%CI 3.05-6.03, I²=95%, p<0.001)

**Figure S3:** Forest plot of studies showing odds ratio of the prevalence rates of chronic GI symptoms in patients with hEDS/HSD, stratified by control group

**Figure S4:** Forest plot of studies with low risk of bias showing prevalence rates of chronic GI symptoms in patients with hEDS/HSD (PP 67.3%, 95% CI, 51.3 - 80.1, I^2^=99.521, p=0.0001).

**Figure S5:** Funnel plot of studies with low risk of bias showing prevalence rates of chronic GI symptoms in patients with hEDS/HSD

**Figure S6:** Forest plot of studies showing prevalence rates of chronic GI symptoms in patients with hEDS/HSD, stratified by geographic region

**Figure S7:** Forest plot of studies showing prevalence rates of heartburn in patients with hEDS/HSD (PP 34.7%, 95%CI 28.3-41.7, I^2^=93.501, p=0.0001)

**Figure S8:** Forest plot of studies showing prevalence rates of abdominal pain in patients with hEDS/HSD (PP 31.3%, 95%CI 20.0-45.4, I^2^=98.521, p=0.0001)

**Figure S9:** Funnel plot of studies showing prevalence rates of abdominal pain in patients with hEDS/HSD

**Figure S10:** Forest plot of studies showing prevalence rates of constipation in patients with hEDS/HSD (PP 21.6%, 95%CI 12.8-34.0, I^2^=98.393, p=0.0001)

**Figure S11:** Funnel plot of studies showing prevalence rates of constipation in patients with hEDS/HSD

**Figure S12:** Forest plot of studies showing prevalence rates of nausea in patients with hEDS/HSD (PP 21.0%, 95%CI 12.4-33.5, I^2^=98.521, p=0.0001)

**Figure S13:** Funnel plot of studies showing prevalence rates of nausea in patients with hEDS/HSD

**Figure S14:** Forest plot of studies showing prevalence rates of dysphagia in patients with hEDS/HSD (PP 18.5%, 95%CI 10.5-30.3, I^2^=98.653, p=0.0001)

**Figure S15:** Funnel plot of studies showing prevalence rates of dysphagia in patients with hEDS/HSD

**Figure S16:** Forest plot of studies showing prevalence rates of postprandial distress in patients with hEDS/HSD (PP 16.6%, 95%CI 7.9-31.7, I^2^=98.629, p=0.0001)

**Figure S17:** Forest plot of studies showing prevalence rates of bloating in patients with hEDS/HSD (PP 11.9%, 95%CI 6.1-21.9, I^2^=97.849, p=0.0001)

**Figure S18:** Funnel plot of studies showing prevalence rates of bloating in patients with hEDS/HSD

**Figure S19:** Forest plot of studies showing prevalence rates of vomiting in patients with hEDS/HSD (PP 10.7%, 95%CI 6.6-16.9, I^2^=96.716, p=0.0001)

**Figure S20:** Forest plot of studies showing prevalence rates of diarrhea in patients with hEDS/HSD (PP 8.2%, 95%CI 3.3-19.2, I^2^=98.423, p=0.0001)

**Figure S21:** Forest plot of studies showing prevalence rates of rectal prolapse in patients with hEDS/HSD (PP 4.8%, 95%CI 1.0-19.6, I^2^=97.577, p=0.0001)

**Figure S22:** Forest plot of studies showing prevalence rates of GERD in patients with hEDS/HSD (PP 41.3%, 95%CI 27.2-57.0, I^2^=94.009, p=0.0001)

**Figure S23:** Funnel plot of studies showing prevalence rates of DGBI in patients with hEDS/HSD

**Figure S24:** Forest plot of studies showing prevalence rates of functional dysphagia in patients with hEDS/HSD (PP 34.2%, 95%CI 25.7-43.8, I^2^=95.159, p=0.0001)

**Figure S25:** Forest plot of studies showing prevalence rates of functional dyspepsia in patients with hEDS/HSD (PP 31.8%, 95%CI 19.0-48.2, I^2^=98.683, p=0.0001)

**Figure S26:** Forest plot of studies showing prevalence rates of functional heartburn in patients with hEDS/HSD (PP 29.9%, 95%CI 23.9-36.8, I^2^=90.301, p=0.0001)

**Figure S27:** Forest plot of studies showing prevalence rates of IBS in patients with hEDS/HSD (PP 23.3%, 95%CI 12.9-38.3, I^2^=99.507, p=0.0001)

**Figure S28:** Funnel plot of studies showing prevalence rates of IBS in patients with hEDS/HSD

**Figure S29:** Forest plot of studies showing prevalence rates of functional constipation in patients with hEDS/HSD (PP 19.3%, 95%CI 10.3-33.1, I^2^=97.232, p=0.0001)

**Figure S30:** Forest plot of studies showing prevalence rates of fecal incontinence in patients with hEDS/HSD (PP 15.8%, 95%CI 12.4-19.8, I^2^=55.563, p=0.105)

**Figure S31:** Forest plot of studies showing prevalence rates of belching disorders in patients with hEDS/HSD (PP 15.2%, 95%CI 8.8-25.0, I^2^=96.162, p=0.0001)

**Figure S32:** Forest plot of studies showing prevalence rates of nausea and vomiting disorders in patients with hEDS/HSD (PP 14.4%, 95%CI 8.6-23.2, I^2^=95.602, p=0.0001)

**Figure S33:** Forest plot of studies showing prevalence rates of rumination in patients with hEDS/HSD (PP 14.1%, 95%CI 4.9-34.2, I^2^=98.995, p=0.0001)

**Figure S34:** Forest plot of studies showing prevalence rates of CVS in patients with hEDS/HSD (PP 13.4%, 95%CI 7.7-22.1, I^2^=96.166, p=0.0001)

**Figure S35:** Forest plot of studies showing prevalence rates of functional bloating in patients with hEDS/HSD (PP 7.8%, 95%CI 2.8-19.9, I^2^=96.668, p=0.0001)

**Figure S36:** Forest plot of studies showing prevalence rates of functional chest pain in patients with hEDS/HSD (PP 7.6%, 95%CI 3.0-17.7, I^2^=97.238, p=0.0001)

**Figure S37:** Forest plot of studies showing prevalence rates of unspecified bowel disorder in patients with hEDS/HSD (PP 7.5%, 95%CI 2.2-22.6, I^2^=97.397, p=0.0001)

**Figure S38:** Forest plot of studies showing prevalence rates of functional diarrhea in patients with hEDS/HSD (PP 5.0%, 95%CI 1.6-14.8, I^2^=96.419, p=0.0001)

**Figure S39:** Forest plot of studies showing prevalence rates of globus in patients with hEDS/HSD (PP 2.1%, 95%CI 1.7-2.7, I^2^=0.0001, p=0.595)

**Figure S40:** Forest plot of studies showing prevalence rates of functional biliary disorders in patients with hEDS/HSD (PP 1.2%, 95%CI 0.7-2.0, I^2^=0.0001, p=0.814)

**Figure S41:** Forest plot of studies showing prevalence rates of chronic fatigue in patients with hEDS/HSD (PP 49%, 95%CI 34.6-63.6, I^2^=93.899, p=0.0001)

**Figure S42:** Forest plot of studies showing prevalence rates of migraine in patients with hEDS/HSD (PP 38.2%, 95%CI 19.9-60.5, I^2^=88.309, p=0.0001)

**Figure S43:** Forest plot of studies showing prevalence rates of fibromyalgia in patients with hEDS/HSD (PP 27.9%, 95%CI 16.0-44.0, I^2^=96.34, p=0.0001)

**Figure S44:** Forest plot of studies showing prevalence rates of orthostatic intolerance in patients with hEDS/HSD (PP 35.9%, 95%CI 26.6-46.4, I^2^=96.397, p=0.0001)

**Figure S45:** Forest plot of studies showing prevalence rates of POTS in patients with hEDS/HSD (PP 21.9%, 95%CI 5.2-59.1, I^2^=99.479, p=0.0001)

**Figure S46:** Forest plot of studies showing prevalence of chronic GI symptoms ion patients with hEDS/HSD, stratified by diagnostic criteria

**Figure S47:** Forest plot of studies showing prevalence rates of chronic GI symptoms in patients with hEDS/HSD with an excluded outlier (PP 71.0%, 95% CI, 60.1 - 79.9, I^2^=99.091, p=0.0001).

**Figure S48:** Forest plot of studies showing odds ratio of the prevalence rates of chronic GI symptoms in patients with hEDS/HSD vs mixed controls with an excluded outlier (OR 4.52, 95%CI 3.11-6.55, I²=95%, p<0.001)

**Legend of Tables:**

**Table S1:** PubMed search strategy from University of Queensland librarian

**Table S2:** Studies excluded from the systematic review and meta-analysis with reasoning.

**Table S3:** Subject characteristics, inclusion and exclusion criteria for the studies included in the systematic review and meta-analysis

**Table S4:** Joanna Briggs Institute critical appraisal for assessment of quality of studies included in this systematic review and meta-analysis.

**Table S5:** Newcastle-Ottawa scale for assessment of case-control studies included in this systematic review and meta-analysis

**Table S6:** Hartung-Knapp adjustment calculated values

**Supplementary Figures:**

**Figure S1:** Funnel plot of studies showing prevalence rates of Chronic GI symptoms in patients with hEDS/HSD

**Figure S2:** Forest plot of studies showing odds ratio of the prevalence rates of chronic GI symptoms in patients with hEDS/HSD vs mixed controls (OR 4.17, 95%CI 3.02-5.77, I²=94%, p<0.001)

**Figure S3:** Forest plot of studies showing odds ratio of the prevalence rates of chronic GI symptoms in patients with hEDS/HSD, stratified by control group

**Figure S4:** Forest plot of studies with low risk of bias showing prevalence rates of chronic GI symptoms in patients with hEDS/HSD (PP 67.3%, 95% CI, 51.3 - 80.1, I^2^=99.521, p=0.0001).

**Figure S5:** Funnel plot of studies with low risk of bias showing prevalence rates of chronic GI symptoms in patients with hEDS/HSD

**Figure S6:** Forest plot of studies showing prevalence rates of chronic GI symptoms in patients with hEDS/HSD, stratified by geographic region

**Figure S7:** Forest plot of studies showing prevalence rates of heartburn in patients with hEDS/HSD (PP 34.7%, 95%CI 28.3-41.7, I^2^=93.501, p=0.0001)

**Figure S8:** Forest plot of studies showing prevalence rates of abdominal pain in patients with hEDS/HSD (PP 31.3%, 95%CI 20.0-45.4, I^2^=98.521, p=0.0001)

**Figure S9:** Funnel plot of studies showing prevalence rates of abdominal pain in patients with hEDS/HSD

**Figure S10:** Forest plot of studies showing prevalence rates of constipation in patients with hEDS/HSD (PP 21.6%, 95%CI 12.8-34.0, I^2^=98.393, p=0.0001)

**Figure S11:** Funnel plot of studies showing prevalence rates of constipation in patients with hEDS/HSD

**Figure S12:** Forest plot of studies showing prevalence rates of nausea in patients with hEDS/HSD (PP 21.0%, 95%CI 12.4-33.5, I^2^=98.521, p=0.0001)

**Figure S13:** Funnel plot of studies showing prevalence rates of nausea in patients with hEDS/HSD

**Figure S14:** Forest plot of studies showing prevalence rates of dysphagia in patients with hEDS/HSD (PP 18.5%, 95%CI 10.5-30.3, I^2^=98.653, p=0.0001)

**Figure S15:** Funnel plot of studies showing prevalence rates of dysphagia in patients with hEDS/HSD

**Figure S16:** Forest plot of studies showing prevalence rates of postprandial distress in patients with hEDS/HSD (PP 16.6%, 95%CI 7.9-31.7, I^2^=98.629, p=0.0001)

**Figure S17:** Forest plot of studies showing prevalence rates of bloating in patients with hEDS/HSD (PP 11.9%, 95%CI 6.1-21.9, I^2^=97.849, p=0.0001)

**Figure S18:** Funnel plot of studies showing prevalence rates of bloating in patients with hEDS/HSD

**Figure S19:** Forest plot of studies showing prevalence rates of vomiting in patients with hEDS/HSD (PP 10.7%, 95%CI 6.6-16.9, I^2^=96.716, p=0.0001)

**Figure S20:** Forest plot of studies showing prevalence rates of diarrhea in patients with hEDS/HSD (PP 8.2%, 95%CI 3.3-19.2, I^2^=98.423, p=0.0001)

**Figure S21:** Forest plot of studies showing prevalence rates of rectal prolapse in patients with hEDS/HSD (PP 4.8%, 95%CI 1.0-19.6, I^2^=97.577, p=0.0001)

**Figure S22:** Forest plot of studies showing prevalence rates of GERD in patients with hEDS/HSD (PP 41.3%, 95%CI 27.2-57.0, I^2^=94.009, p=0.0001)

**Figure S23:** Funnel plot of studies showing prevalence rates of DGBI in patients with hEDS/HSD

**Figure S24:** Forest plot of studies showing prevalence rates of functional dysphagia in patients with hEDS/HSD (PP 34.2%, 95%CI 25.7-43.8, I^2^=95.159, p=0.0001)

**Figure S25:** Forest plot of studies showing prevalence rates of functional dyspepsia in patients with hEDS/HSD (PP 31.8%, 95%CI 19.0-48.2, I^2^=98.683, p=0.0001)

**Figure S26:** Forest plot of studies showing prevalence rates of functional heartburn in patients with hEDS/HSD (PP 29.9%, 95%CI 23.9-36.8, I^2^=90.301, p=0.0001)

**Figure S27:** Forest plot of studies showing prevalence rates of IBS in patients with hEDS/HSD (PP 23.3%, 95%CI 12.9-38.3, I^2^=99.507, p=0.0001)

**Figure S28:** Funnel plot of studies showing prevalence rates of IBS in patients with hEDS/HSD

**Figure S29:** Forest plot of studies showing prevalence rates of functional constipation in patients with hEDS/HSD (PP 19.3%, 95%CI 10.3-33.1, I^2^=97.232, p=0.0001)

**Figure S30:** Forest plot of studies showing prevalence rates of fecal incontinence in patients with hEDS/HSD (PP 15.8%, 95%CI 12.4-19.8, I^2^=55.563, p=0.105)

**Figure S31:** Forest plot of studies showing prevalence rates of belching disorders in patients with hEDS/HSD (PP 15.2%, 95%CI 8.8-25.0, I^2^=96.162, p=0.0001)

**Figure S32:** Forest plot of studies showing prevalence rates of nausea and vomiting disorders in patients with hEDS/HSD (PP 14.4%, 95%CI 8.6-23.2, I^2^=95.602, p=0.0001)

**Figure S33:** Forest plot of studies showing prevalence rates of rumination in patients with hEDS/HSD (PP 14.1%, 95%CI 4.9-34.2, I^2^=98.995, p=0.0001)

**Figure S34:** Forest plot of studies showing prevalence rates of CVS in patients with hEDS/HSD (PP 13.4%, 95%CI 7.7-22.1, I^2^=96.166, p=0.0001)

**Figure S35:** Forest plot of studies showing prevalence rates of functional bloating in patients with hEDS/HSD (PP 7.8%, 95%CI 2.8-19.9, I^2^=96.668, p=0.0001)

**Figure S36:** Forest plot of studies showing prevalence rates of functional chest pain in patients with hEDS/HSD (PP 7.6%, 95%CI 3.0-17.7, I^2^=97.238, p=0.0001)

**Figure S37:** Forest plot of studies showing prevalence rates of unspecified bowel disorder in patients with hEDS/HSD (PP 7.5%, 95%CI 2.2-22.6, I^2^=97.397, p=0.0001)

**Figure S38:** Forest plot of studies showing prevalence rates of functional diarrhea in patients with hEDS/HSD (PP 5.0%, 95%CI 1.6-14.8, I^2^=96.419, p=0.0001)

**Figure S39:** Forest plot of studies showing prevalence rates of globus in patients with hEDS/HSD (PP 2.1%, 95%CI 1.7-2.7, I^2^=0.0001, p=0.595)

**Figure S40:** Forest plot of studies showing prevalence rates of functional biliary disorders in patients with hEDS/HSD (PP 1.2%, 95%CI 0.7-2.0, I^2^=0.0001, p=0.814)

**Figure S41:** Forest plot of studies showing prevalence rates of chronic fatigue in patients with hEDS/HSD (PP 49%, 95%CI 34.6-63.6, I^2^=93.899, p=0.0001)

**Figure S42:** Forest plot of studies showing prevalence rates of migraine in patients with hEDS/HSD (PP 38.2%, 95%CI 19.9-60.5, I^2^=88.309, p=0.0001)

**Figure S43:** Forest plot of studies showing prevalence rates of fibromyalgia in patients with hEDS/HSD (PP 27.9%, 95%CI 16.0-44.0, I^2^=96.34, p=0.0001)

**Figure S44:** Forest plot of studies showing prevalence rates of orthostatic intolerance in patients with hEDS/HSD (PP 35.9%, 95%CI 26.6-46.4, I^2^=96.397, p=0.0001)

**Figure S45:** Forest plot of studies showing prevalence rates of POTS in patients with hEDS/HSD (PP 21.9%, 95%CI 5.2-59.1, I^2^=99.479, p=0.0001)

**Figure S46:** Forest plot of studies showing prevalence of chronic GI symptoms ion patients with hEDS/HSD, stratified by diagnostic criteria

**Figure S47:** Forest plot of studies showing prevalence rates of chronic GI symptoms in patients with hEDS/HSD with an excluded outlier (PP 71.0%, 95% CI, 60.1 - 79.9, I^2^=99.091, p=0.0001).

**Figure S48:** Forest plot of studies showing odds ratio of the prevalence rates of chronic GI symptoms in patients with hEDS/HSD vs mixed controls with an excluded outlier (OR 4.52, 95%CI 3.11-6.55, I²=95%, p<0.001)

**Supplementary Tables:**

**Table S1:** PubMed search strategy from University of Queensland librarian

1. gut-brain
2. brain-gut axis
3. Irritable bowel syndrome
4. "Functional dyspepsia"
5. "Functional constipation"
6. "Reflux hypersensitivity"
7. "Chronic bowel dysfunction"
8. "Functional bowel disorder"
9. "Functional abdominal pain disorder"
10. "Chronic nausea and vomiting syndrome"
11. "Motility disorder"
12. "Functional chest pain"
13. "Functional heartburn"
14. Globus
15. "Functional dysphagia"
16. "Postprandial distress syndrome"
17. Epigastric pain syndrome
18. Belching disorder
19. cyclic vomiting syndrome
20. "functional diarrhea"
21. "functional abdominal bloating"
22. "biliary pain"
23. "Functional gallbladder disorder"
24. "functional sphincter of Oddi disorder"
25. faecal incontinence
26. "functional defecation disorder"
27. "functional anorectal pain"
28. "gastrointestinal diseases"[MeSH Terms]
29. "Joint hypermobility"
30. "hypermobility syndrome"
31. "hypermobility spectrum disorder"
32. Ehlers-Danlos syndrome [MeSH Terms]
33. (1 OR 2 OR 3 OR 4 OR 5 OR 6 OR 7 OR 8 OR 9 OR 10 OR 11 OR 12 OR 13 OR 14 OR 15 OR 16 OR 17 OR 18 OR 19 OR 20 OR 21 OR 22 OR 23 OR 24 OR 25 OR 26 OR 27) AND (29 OR 30 OR 31 OR 32)
34. 28 AND (29 OR 30 OR 31 OR 32)
35. “dyspepsia”
36. “constipation”
37. “reflux”
38. “abdominal pain”
39. “nausea”
40. “vomiting”
41. “heartburn”
42. “dysphagia”
43. “postprandial distress”
44. “epigastric pain”
45. “belching”
46. “diarrhea”
47. “bloating”
48. “gastrointestinal”
49. (35 OR 36 OR 37 OR 38 OR 39 OR 40 OR 41 OR 42 OR 43 OR 44 OR 45 OR 46 OR 47 OR 48) AND (29 OR 30 OR 31 OR 32)

Limit: 01/1960-09/2024

Filters applied: MEDLINE

PubMed: 866

**Table S2:** Studies excluded from the systematic review and meta-analysis with reasoning.

| Study | Reason for exclusion |
| --- | --- |
| Fikree et al., 2015^1^ | Data irrelevant to the research question |
| Zweig et al., 2018^2^ | Data irrelevant to the research question |
| Carbone et al., 2021^3^ | Data irrelevant to the research question |
| Kovacic et al., 2014^4^ | Data irrelevant to the research question |
| Carbone et al., 2020^5^ | Data irrelevant to the research question |
| Quitadamo et al., 2021^6^ | Data irrelevant to the research question |
| Shulman et al., 2020^7^ | Data irrelevant to the research question |
| Fikree et al., 2014^8^ | Gastrointestinal symptoms were an inclusion criterion |
| Zarate et al., 2010^9^ | Gastrointestinal symptoms were an inclusion criterion |
| Loganathan et al., 2024^10^ | Gastrointestinal symptoms were an inclusion criterion |
| Khorasgani et al., 2020^11^ | Gastrointestinal symptoms were an inclusion criterion |
| Saps et al., 2018^12^ | Gastrointestinal symptoms were an inclusion criterion |
| Sahin et al., 2023^13^ | Gastrointestinal symptoms were an inclusion criterion |
| Sood et al., 2024^14^ | Gastrointestinal symptoms were an inclusion criterion |
| Fikree et al., 2017^15^ | Gastrointestinal symptoms were an inclusion criterion |
| Kishore et al., 2013^16^ | Gastrointestinal symptoms were an inclusion criterion |
| Ritelli et al., 2020^17^ | No individual GI symptoms reported |
| Frank et al., 2018^18^ | No individual GI symptoms reported |

**Table S3:** Subject characteristics, inclusion and exclusion criteria for the studies included in the systematic review and meta-analysis.

| **No** | **Author** | **Mean Age, years (SD)** | **Females, n (%)** | **GI symptoms** | **Clinical diagnostic criteria (hEDS/HSD)** | **Clinical diagnostic criteria (GI pathology)** | **Inclusions** | **Exclusion criteria** |
| --- | --- | --- | --- | --- | --- | --- | --- | --- |
| 1 | Castori et al. (2010)^19^ | 34.8 (NA) | 18 (85.7) | Assessed by clinician | Brighton criteria, Villefranche criteria, Beighton score | Clinical diagnosis | Hypermobility type Ehlers-Danlos syndrome patients | Other connective tissue and muscle disorders, developmental delay, atrophic/hemosiderotic scars, molluscoid pseudotumors, subcutaneous spheroids |
| 2 | Zeitoun et al.^20^ | 35.0 (14.7) | 123 (91.8) | Survey | Beighton scale, Villefranche criteria | Rome III, Kess score | Ehlers-Danlos syndrome patients | NA |
| 3 | Nelson et al.^21^ | 29.6 (14.8) | NA | Medical records | Brighton criteria, Villefranche criteria | Clinical diagnosis | Ehlers-Danlos syndrome patients | Patients with family history of EDS but without personal diagnosis, patients with other connective tissue dysplasia |
| 4 | Brooks et al.^22^ | 36.6 (18.1) | 1691 (84.3) | Medical records | Clinical diagnosis | Clinical diagnosis | Ehlers-Danlos syndrome patients | NA |
| 5 | Hakim et al.^23^ | NA | 170 (100) | Assessed by clinician | Brighton criteria | Clinical diagnosis | Hypermobility clinic patients | NA |
| 6 | Alomari et al.^24^ | 32.3 (NA) | 198 (90.8) | Medical records | 2017 international criteria | Rome III-IV | Hypermobility type Ehlers-Danlos syndrome patients | Organic GI diseases, personal history of GI surgery, patients who did not meet the 2017 international criteria for hEDS, patients with other causes for gut dysmotility |
| 7 | Castori et al. (2011)^25^ | 31.6 (NA) | 44 (88) | Assessed by clinician | Brighton criteria, Villefranche criteria | Clinical diagnosis | Joint hypermobility and hypermobility type Ehlers-Danlos syndrome patients | Other heritable connective tissue disorders, patients with doubtful or incomplete diagnosis |
| 8 | Inayet et al.^26^ | 24 (NA) | 39 (86.7) | Validated questionnaire | Clinical diagnosis | Rome IV | Marfan syndrome and hypermobile Ehlers-Danlos syndrome patients | Organic GI diseases |
| 9 | Lam et al.^27^ | 39 (NA) | 580 (96.2) | Survey | Clinical diagnosis | Rome IV | Hypermobility type Ehlers-Danlos syndrome patients | Inconsistent responders, other subtypes of EDS, organic GI pathology |
| 10 | Wang et al.^28^ | 38.2 (16.5) | 40 (58.8) | Medical records | 2017 international criteria | Clinical diagnosis | Loeys-Dietz syndrome and vascular Ehlers-Danlos syndrome patients | Other subtypes or indeterminate subtype of EDS, patients deemed to not have a disorder based on clinical review/genetic testing |
| 11 | Fikree et al. (2016)^29^ | 21 (NA) | 48 (64.9) | Validated questionnaire | Brighton criteria, Villefranche criteria | BDQ, Rome III | University students screening positive for hypermobility type Ehlers-Danlos syndrome | University students screening negative for hypermobility type Ehlers-Danlos syndrome |
| 12 | Dhingra et al.^30^ | 29.6 (16.3) | 3042 (70.8) | Medical records | Villefranche criteria | Clinical diagnosis | Ehlers-Danlos syndrome patients | NA |
| 13 | Wasim et al.^31^ | 36.1 (14.4) | 334 (85.4) | Medical records | 2017 international criteria | Clinical diagnosis | Joint hypermobility and hypermobility type Ehlers-Danlos syndrome patients | Other connective tissue pathology |
| 14 | Nee et al.^32^ | 40.9 (13.2) | 1691 (93.9) | Survey | Genetic testing, clinical diagnosis | Rome III | Marfan syndrome and Ehlers-Danlos syndrome patients | NA |
| 15 | Tai et al.^33^ | 39.1 (12.8) | 593 (96.3) | Survey | Clinical diagnosis | Rome IV | Hypermobility type Ehlers-Danlos syndrome patients | Organic GI disease |
| 16 | Silvernale et al.^34^ | 44.3 (NA) | 183 (94.8) | Survey | Clinical diagnosis | Rome IV | Hypermobility type Ehlers-Danlos syndrome patients | NA |
| 27 | Zloof et al.^35^ | 17.2 (0.5) | 2133 (45.5) | Assessed by clinician | Beighton score, Brighton criteria | Rome II-IV | Hypermobility type Ehlers-Danlos syndrome adolescent patients | Other connective tissue disorders |
| 18 | Leganger et al.^36^ | 31 (NA) | 1002 (76.0) | Medical records | Clinical diagnosis | Clinical diagnosis | Ehlers-Danlos syndrome patients | None |
| 19 | Solomon et al.^37^ | NA | NA | Medical records | Clinical diagnosis | Rome IV | Children with POTS and joint hypermobility syndrome or Ehlers-Danlos syndrome | NA |

SD – standard deviation; hEDS – hypermobility type Ehlers-Danlos syndrome; HSD – hypermobility spectrum disorders; GI – gastrointestinal; NA – not available

**Table S4:** Joanna Briggs Institute critical appraisal for assessment of quality of prevalence studies included in this systematic review and meta-analysis

| No | Author | 1) sample  frame  appropriate  to address  target pop  (DGBI  subjects) | 2)  participants  sampled in an  appropriate  way | 3) sample  size  adequate | 4) study  subjects  and  setting  details | 5) data  analysis  conducted  with  sufficient  coverage  (response  rate same  between  categories) | 6) valid methods  for identifying  condition  (ROME) | 7) condition measured in  standard reliable way for  all participants  (questionnaire/clinical  diagnosis) | 8)  appropriate  statistical  analysis | 9)  response  rate  adequate  (>80%) | JBI  total | JBI  total  % |
| --- | --- | --- | --- | --- | --- | --- | --- | --- | --- | --- | --- | --- |
| 1 | Castori et al. (2010) | 1 | 1 | U | 1 | 1 | U | 1 | N | 1 | 6 | 66.7 |
| 2 | Zeitoun et al. | 1 | 1 | U | 1 | 1 | 1 | 1 | 1 | N | 7 | 77.8 |
| 3 | Nelson et al. | 1 | 1 | 1 | 1 | 1 | U | 1 | 1 | 1 | 8 | 88.9 |
| 4 | Brooks et al. | 1 | 1 | 1 | 1 | 1 | U | 1 | 1 | 1 | 8 | 88.9 |
| 5 | Hakim et al. | 1 | 1 | U | 1 | N | U | 1 | N | U | 4 | 44.4 |
| 6 | Alomari et al. | 1 | 1 | 1 | 1 | 1 | 1 | 1 | 1 | 1 | 9 | 100 |
| 7 | Castori et al. (2011) | 1 | 1 | N | N | 1 | U | 1 | 1 | N | 5 | 55.6 |
| 8 | Inayet et al. | 1 | 1 | N | 1 | 1 | U | 1 | 1 | 1 | 7 | 77.8 |
| 9 | Lam et al. | 1 | 1 | 1 | 1 | 1 | U | 1 | 1 | N | 7 | 77.8 |
| 10 | Wang et al. | 1 | 1 | N | 1 | 1 | U | 1 | 1 | 1 | 7 | 77.8 |
| 11 | Fikree et al. (2016) | 1 | 1 | N | 1 | 1 | 1 | 1 | 1 | N | 7 | 77.8 |
| 12 | Dhingra et al. | 1 | 1 | 1 | 1 | 1 | U | 1 | 1 | 1 | 8 | 88.9 |
| 13 | Wasim et al. | 1 | 1 | 1 | 1 | 1 | U | 1 | 1 | 1 | 8 | 88.9 |
| 14 | Nee et al. | 1 | 1 | 1 | 1 | 1 | 1 | 1 | 1 | N | 8 | 88.9 |
| 15 | Tai et al. | 1 | 1 | 1 | 1 | 1 | U | 1 | 1 | N | 7 | 77.8 |
| 16 | Silvernale et al. | 1 | 1 | U | 1 | N | U | 1 | 1 | N | 5 | 55.6 |
| 17 | Zloof et al. | 1 | 1 | 1 | 1 | 1 | 1 | 1 | 1 | 1 | 9 | 100 |
| 18 | Leganger et al. | 1 | 1 | 1 | 1 | 1 | U | 1 | 1 | 1 | 8 | 88.9 |
| 19 | Solomon et al. | 1 | 1 | N | N | U | 1 | 1 | U | U | 4 | 44.4 |

**Table S5:** Newcastle-Ottawa scale for assessment of case-control studies included in this systematic review and meta-analysis

| No | Author | Case definition | Representativeness | Selection of controls | Definition of controls | Comparability | Ascertainment of exposure | Same method for cases and controls | Non-response rate | Total |
| --- | --- | --- | --- | --- | --- | --- | --- | --- | --- | --- |
| 1 | Brooks et al. | b | a | b | a | ab | a | a | a | 7 |
| 2 | Inayet et al. | b | a | b | a | ab | a | a | a | 7 |
| 3 | Lam et al. | b | a | a | a | ab | a | a | a | 8 |
| 4 | Fikree et al. | a | a | a | a | ab | a | a | a | 9 |
| 5 | Dhingra et al. | b | a | b | a | ab | a | a | a | 7 |
| 6 | Zloof et al. | a | a | a | a | ab | a | a | a | 9 |
| 7 | Leganger et al. | b | a | a | a | ab | a | a | a | 8 |

**Table S6:** Hartung-Knapp adjustment calculated values

|  | Number of studies, n | Number of eligible participants, n | Pooled prevalence, % (95% CI) |
| --- | --- | --- | --- |
| Prevalence of functional dysphagia in patients with hEDS/HSD | 4 | 3,068 | 34.2 (19.9 to 52.1) |
| Prevalence of functional heartburn in patients with hEDS/HSD | 4 | 3,068 | 29.9 (18.4 to 44.8) |
| Prevalence of functional constipation in patients with hEDS/HSD | 5 | 3,202 | 19.3 (6.0 to 47.3) |
| Prevalence of fecal incontinence in patients with hEDS/HSD | 3 | 1,264 | 15.8 (7.1 to 31.6) |
| Prevalence of belching disorders in patients with hEDS/HSD | 4 | 3,068 | 15.2 (6.1 to 33.0) |
| Prevalence of nausea and vomiting disorders in patients with hEDS/HSD | 4 | 3,068 | 14.4 (6.1 to 30.4) |
| Prevalence of rumination in patients with hEDS/HSD | 4 | 3,068 | 14.1 (2.2 to 54.6) |
| Prevalence of CVS in patients with hEDS/HSD | 3 | 3,023 | 13.4 (3.9 to 37.0) |
| Prevalence of functional bloating in patients with hEDS/HSD | 4 | 3,068 | 7.8 (1.0 to 41.2) |
| Prevalence of functional chest pain in patients with hEDS/HSD | 4 | 3,068 | 7.6 (1.7 to 28.2) |
| Prevalence of unspecified bowel disorder in patients with hEDS/HSD | 4 | 3,068 | 7.5 (0.1 to 88.8) |
| Prevalence of functional diarrhea in patients with hEDS/HSD | 4 | 3,068 | 5.0 (0.4 to 40.6) |
| Prevalence of globus in patients with hEDS/HSD | 4 | 3,068 | 2.1 (1.4 to 3.2) |
| Prevalence of functional biliary disorders in patients with hEDS/HSD | 3 | 1,264 | 1.2 (0.4 to 3.6) |
| Prevalence of chronic fatigue in patients with hEDS/HSD | 5 | 1,442 | 49.0 (13.1 to 86.0) |
| Prevalence of GERD in patients with hEDS/HSD | 5 | 2,370 | 41.3 (18.1 to 69.1) |
| Prevalence of migraine in patients with hEDS/HSD | 3 | 776 | 38.2 (3.5 to 91.2) |
| Prevalence of POTS in patients with hEDS/HSD | 3 | 1,264 | 21.9 (0.8 to 91.1) |
| Prevalence of rectal prolapse in patients with hEDS/HSD | 3 | 2,709 | 4.8 (0.2 to 61.9) |
| Odds ratio of chronic GI symptom prevalence in patients with hEDS/HSD vs healthy controls | 4 | 6,682 (1,669,112) | 5.05 (0.4 to 59.3) |

CI – confidence interval; hEDS – hypermobility type Ehlers-Danlos syndrome; HSD – hypermobility spectrum disorders; CVS – cyclic vomiting syndrome; GERD – gastroesophageal reflux disease; POTS – postural orthostatic tachycardia syndrome; GI - gastrointestinal

**References:**

1.	Fikree A, Aktar R, Grahame R, et al. Functional gastrointestinal disorders are associated with the joint hypermobility syndrome in secondary care: a case-control study. Neurogastroenterol Motil 2015;27:569-79.

2.	Zweig A, Schindler V, Becker AS, et al. Higher prevalence of joint hypermobility in constipation predominant irritable bowel syndrome. Neurogastroenterol Motil 2018;30:e13353.

3.	Carbone F, Goelen N, Fikree A, et al. Impact of joint hypermobility syndrome on gastric accommodation and nutrient tolerance in functional dyspepsia. Neurogastroenterol Motil 2021;33:e14086.

4.	Kovacic K, Chelimsky TC, Sood MR, et al. Joint hypermobility: a common association with complex functional gastrointestinal disorders. J Pediatr 2014;165:973-8.

5.	Carbone F, Fikree A, Aziz Q, et al. Joint Hypermobility Syndrome in Patients With Functional Dyspepsia. Clin Transl Gastroenterol 2020;11:e00220.

6.	Quitadamo P, Thapar N, Nardo GD, et al. Is there a relationship between joint hypermobility and gastrointestinal disorders in children? Turk J Pediatr 2021;63:307-313.

7.	Shulman RJ, Self MM, Czyzewski DI, et al. The Prevalence of Hypermobility in Children with Irritable Bowel Syndrome and Functional Abdominal Pain Is Similar to that in Healthy Children. J Pediatr 2020;222:134-140 e2.

8.	Fikree A, Grahame R, Aktar R, et al. A prospective evaluation of undiagnosed joint hypermobility syndrome in patients with gastrointestinal symptoms. Clin Gastroenterol Hepatol 2014;12:1680-87 e2.

9.	Zarate N, Farmer AD, Grahame R, et al. Unexplained gastrointestinal symptoms and joint hypermobility: is connective tissue the missing link? Neurogastroenterol Motil 2010;22:252-e78.

10.	Loganathan P, Herlihy D, Gajendran M, et al. The spectrum of gastrointestinal functional bowel disorders in joint hypermobility syndrome and in an academic referral center. J Investig Med 2024;72:162-168.

11.	Khorasgani SMF, Ramezani N, Varnousfaderani NE. Joint hypermobility in children with and without functional constipation. J Res Med Sci 2020;25:28.

12.	Saps M, Blom PJJ, Velasco-Benitez CA, et al. Functional Gastrointestinal Disorders and Joint Hypermobility: A School-based Study. J Pediatr Gastroenterol Nutr 2018;66:387-390.

13.	Sahin NU, Sahin N, Kilic M. Effect of comorbid benign joint hypermobility and juvenile fibromyalgia syndromes on pediatric functional gastrointestinal disorders. Postgrad Med 2023;135:386-393.

14.	Sood V, Kaul A, El-Chammas KI, et al. High prevalence of gastrointestinal disorders in a large cohort of patients with joint hypermobility. J Pediatr Gastroenterol Nutr 2024;79:42-47.

15.	Fikree A, Aziz Q, Sifrim D. Mechanisms underlying reflux symptoms and dysphagia in patients with joint hypermobility syndrome, with and without postural tachycardia syndrome. Neurogastroenterol Motil 2017;29.

16.	Kishore S. Gastrointestinal manifestations of ehler-danlos syndrome type III (hypermobility type) : A paediatric cohort. Digestive Disease Week 2013, DDW 2013. Volume 144:5. Orlando, FL, United States: Gastroenterology, 2013:S610.

17.	Ritelli M, Venturini M, Cinquina V, et al. Multisystemic manifestations in a cohort of 75 classical Ehlers-Danlos syndrome patients: natural history and nosological perspectives. Orphanet J Rare Dis 2020;15:197.

18.	Frank M, Adham S, Zinzindohoue F, et al. Natural history of gastrointestinal manifestations in vascular Ehlers-Danlos syndrome: A 17-year retrospective review. J Gastroenterol Hepatol 2019;34:857-863.

19.	Castori M, Camerota F, Celletti C, et al. Natural history and manifestations of the hypermobility type Ehlers-Danlos syndrome: a pilot study on 21 patients. Am J Med Genet A 2010;152A:556-64.

20.	Zeitoun JD, Lefevre JH, de Parades V, et al. Functional digestive symptoms and quality of life in patients with Ehlers-Danlos syndromes: results of a national cohort study on 134 patients. PLoS One 2013;8:e80321.

21.	Nelson AD, Mouchli MA, Valentin N, et al. Ehlers Danlos syndrome and gastrointestinal manifestations: a 20-year experience at Mayo Clinic. Neurogastroenterol Motil 2015;27:1657-66.

22.	Brooks RS, Grady J, Lowder TW, et al. Prevalence of gastrointestinal, cardiovascular, autonomic and allergic manifestations in hospitalized patients with Ehlers-Danlos syndrome: a case-control study. Rheumatology (Oxford) 2021;60:4272-4280.

23.	Hakim AJ, Grahame R. Non-musculoskeletal symptoms in joint hypermobility syndrome. Indirect evidence for autonomic dysfunction? Rheumatology (Oxford) 2004;43:1194-5.

24.	Alomari M, Hitawala A, Chadalavada P, et al. Prevalence and Predictors of Gastrointestinal Dysmotility in Patients with Hypermobile Ehlers-Danlos Syndrome: A Tertiary Care Center Experience. Cureus 2020;12:e7881.

25.	Castori M, Sperduti I, Celletti C, et al. Symptom and joint mobility progression in the joint hypermobility syndrome (Ehlers-Danlos syndrome, hypermobility type). Clin Exp Rheumatol 2011;29:998-1005.

26.	Inayet N, Hayat JO, Kaul A, et al. Gastrointestinal Symptoms in Marfan Syndrome and Hypermobile Ehlers-Danlos Syndrome. Gastroenterol Res Pract 2018;2018:4854701.

27.	Lam CY, Palsson OS, Whitehead WE, et al. Rome IV Functional Gastrointestinal Disorders and Health Impairment in Subjects With Hypermobility Spectrum Disorders or Hypermobile Ehlers-Danlos Syndrome. Clin Gastroenterol Hepatol 2021;19:277-287 e3.

28.	Wang XJ, Babameto M, Babovic-Vuksanovic D, et al. Audit of Gastrointestinal Manifestations in Patients with Loeys-Dietz Syndrome and Vascular Ehlers-Danlos Syndrome. Dig Dis Sci 2021;66:1142-1152.

29.	Fikree A, Aktar R, Morris JK, et al. The association between Ehlers-Danlos syndrome-hypermobility type and gastrointestinal symptoms in university students: a cross-sectional study. Neurogastroenterol Motil 2017;29.

30.	Dhingra R, Bascom R, Thompson E, et al. Gastrointestinal medication burden among persons with the Ehlers-Danlos syndromes. Neurogastroenterol Motil 2021;33:e14077.

31.	Wasim S, Suddaby JS, Parikh M, et al. Pain and gastrointestinal dysfunction are significant associations with psychiatric disorders in patients with Ehlers-Danlos syndrome and hypermobility spectrum disorders: a retrospective study. Rheumatol Int 2019;39:1241-1248.

32.	Nee J, Kilaru S, Kelley J, et al. Prevalence of Functional GI Diseases and Pelvic Floor Symptoms in Marfan Syndrome and Ehlers-Danlos Syndrome: A National Cohort Study. J Clin Gastroenterol 2019;53:653-659.

33.	Tai FWD, Palsson OS, Lam CY, et al. Functional gastrointestinal disorders are increased in joint hypermobility-related disorders with concomitant postural orthostatic tachycardia syndrome. Neurogastroenterol Motil 2020;32:e13975.

34.	Silvernale C, Garcia-Fischer I, Staller K. Relationship Between Psychological Trauma and Irritable Bowel Syndrome and Functional Dyspepsia in a Joint Hypermobility Syndrome/Ehlers-Danlos Syndrome Patient Population. Dig Dis Sci 2024;69:870-875.

35.	Zloof Y, Peretz L, Braun M, et al. Hypermobility spectrum disorders and irritable bowel syndrome: A nationwide study of 1.6 million adolescents. J Gastroenterol Hepatol 2023;38:2076-2082.

36.	Leganger J, Fonnes S, Kulas Soborg ML, et al. The most common comorbidities in patients with Ehlers-Danlos syndrome: a 15-year nationwide population-based cohort study. Disabil Rehabil 2022;44:189-193.

37.	Solomon V. Presence of functional gastrointestinal disorders (FGIDs) in pediatric patients with ehlers-danlos syndrome or joint hypermobility syndrome and postural orthostatic tachycardia syndrome. North American Society for Pediatric Gastroenterology, Hepatology and Nutrition Annual Meeting, NASPGHAN 2021. Volume 73:1. Virtual: Journal of Pediatric Gastroenterology and Nutrition, 2021:S446-S437.
